# Unlocking body-surface physiological evolution via IR-temperature dual sensing with single chalcogenide fiber

**DOI:** 10.1038/s41377-025-01840-y

**Published:** 2025-04-25

**Authors:** Yanqing Fu, Shiliang Kang, Gangjie Zhou, Xinxiang Huang, Linling Tan, Chengwei Gao, Shixun Dai, Changgui Lin

**Affiliations:** 1 Laboratory of Infrared Materials and Devices, The Research Institute of Advanced Technologies, Ningbo University, Ningbo, 315211 China; 2 Zhejiang Key Laboratory of Advanced Optical Functional Materials and Devices, Ningbo, 315211 China; 3 Engineering Research Center for Advanced Infrared Photoelectric Materials and Devices of Zhejiang Province, Ningbo, 315211 China

**Keywords:** Mid-infrared photonics, Optical sensors

## Abstract

Improvements to body-surface physiological monitoring ability including real-time, accuracy and integration, are essential to meet the expansive demands for personal healthcare. As part of this, simultaneous monitoring of sweat metabolites and body temperature offers an exciting path to maximizing diagnostic precision and minimizing morbidity rates. Herein, we report a high-performance biomarker-temperature sensor made of a single As_3_Se_5_Te_2_ chalcogenide glass fiber to monitor physiology evolution on body-surface. The sensor integrates efficient thermal resistance and fiber evanescent wave effects, permitting the independent sensing of temperature and biomarkers with an ultrahigh temperature coefficient of resistance (−5.84% K^–1^), rapid temperature response (0.3 s) and excellent IR sensing sensitivity. Moreover, by attaching a fiber to the wrist, we demonstrate simultaneous observation of both sweat metabolite (urea and lactate) and temperature changes during exercise. This illuminating sensing method will provide crucial capabilities in physiological monitoring and pave the way for advanced personalized diagnostic.

## Abstract (web summary)

The article introduces a single chalcogenide glass fiber enables simultaneous and efficient sensing of body-surface temperature and sweat biomarkers for real-time physiological monitoring.

## Graphical abstract

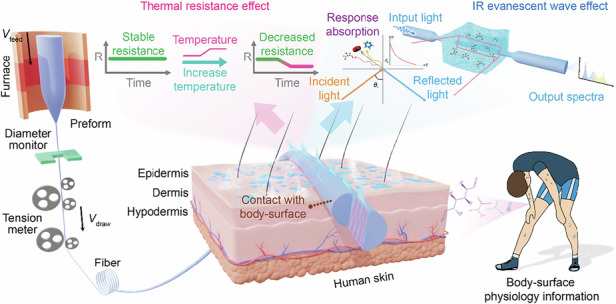

## Introduction

Chronic diseases significantly contribute to global disease burden and have emerged as major public health concerns^1–3^. Early diagnosis of these diseases and accurate, continuous health monitoring are crucial for guiding treatments that reduce morbidity rates and enhance the life quality of patients^4,5^. Skin is the largest organ of the human body, playing a significant role in protecting the body from external substances and serving as a diagnostic interface rich in vital physical, chemical, and biological information related to human health^6,7^. Body-surface physiological monitoring technology has a non-invasive nature, real-time capabilities, and diversity of information, and hence, has recently emerged as an ideal candidate for point-of-care health monitoring^8,9^.

Temperature is a crucial physiological parameter affecting almost all metabolic activities. A relatively constant body temperature is an essential indicator of overall health and bodily function^10^. Thus far, various sensors have been developed to monitor body temperature^11–13^. However, a single temperature parameter is insufficient for the comprehensive evaluation of the health condition or for the prediction and diagnosis of diseases. Therefore, there is a need to develop multifunctional sensors to monitor multiple health signals. Sweat, as a biofluid, contains a wealth of chemical information (e.g., concentrations of glucose, lactic acid, and urea) that could potentially indicate the deeper biomolecular state of the body. Importantly, sweat is readily accessible using non-invasive techniques^14,15^. Current primary sweat detection technologies include electrochemical^16–18^, optical (e.g., fluorescence sensing and colorimetric methods), and fiber Bragg grating methods^19–22^. Sweat sensors based on electrochemical sensing methods have high sensitivity and selectivity; however, their complicated fabrication technologies and procedures, as well as signal crosstalk caused by temperature, pH, and salinity variations, poses challenges for their further development^23,24^. In contrast, sweat sensors based on optical methods feature a low cost, long lifespan, and ease of miniaturization; however, they are susceptible to interference from ambient light and may have poor selectivity owing to the presence of other sweat components^25,26^. Fiber Bragg grating method exhibits excellent sensing sensitivity, but the degradation and detachment of surface functionalization layers reduce its effectiveness and limit long-term use^27,28^. Therefore, there is an urgent need to develop an emerging body-surface physiology sensing strategy to achieve the precise detection of skin temperature and sweat biomarkers simultaneously.

Mid-IR fiber evanescent wave spectroscopy (MIR-FEWS) is an emerging technology that allows for the label-free profiling of a substance by examining molecular fragments (such as chemical bonds and functional groups) and measuring their resonant vibrational response to MIR excitation, has recently been employed in the food and chemical industries, in environmental monitoring, and for battery chemistry and medical applications^29–31^. Chalcogenide glasses (ChGs) are considered ideal materials for MIR-FEWS owing to their broad IR transparency range and excellent shaping ability^32^, which facilitate the drawing of fibers and potential applications in wearable sensing. Additionally, the semiconductor property of ChGs make them appealing for the fabrication of resistance temperature sensors. Building on these superior properties, recent studies have explored the temperature-sensing performance of Ge-As-Se-Te^33^ and biochemical sensing performance of As-S, As-Se, Ge-Sb-Se, and Ge-Te-AgI fibers^34–37^. Despite intensive research efforts and advances in ChG-based IR and temperature sensing, their potential for sweat biomarker and temperature sensing has not been thoroughly explored because of conflicting relationships among the thermal stability, IR transmission, and semiconductor properties.

Here, we present dual-parameter fiber-based sensors for monitoring sweat biomarkers and temperature using the ChG As_3_Se_5_Te_2_ (AST). By leveraging the independent thermal resistance and fiber evanescent wave effects within a single AST fiber, we can simultaneously monitor sweat biomarkers (such as glucose, urea, and lactate) and temperature by transducing body surface information into distinct optical and electrical signals. The AST fiber exhibits high temperature coefficient of resistance (TCR) of approximately −5.84% K^−1^, a temperature resolution of 0.2 K, a response time of 0.3 s, and an exceptional sensitivity in detecting sweat biomarkers. Based on the impressive dual-mode sensing properties and independent sweat biomarker and temperature detection capabilities, we demonstrated the potential applications of this fiber in monitoring physiological states during exercise. This novel body-surface monitoring technology has opened up a window of opportunity for the advancement of personalized diagnostics and health condition monitoring.

## Results

### AST fiber fabrication and IR-temperature sensing mechanism

**Fig. 1:**
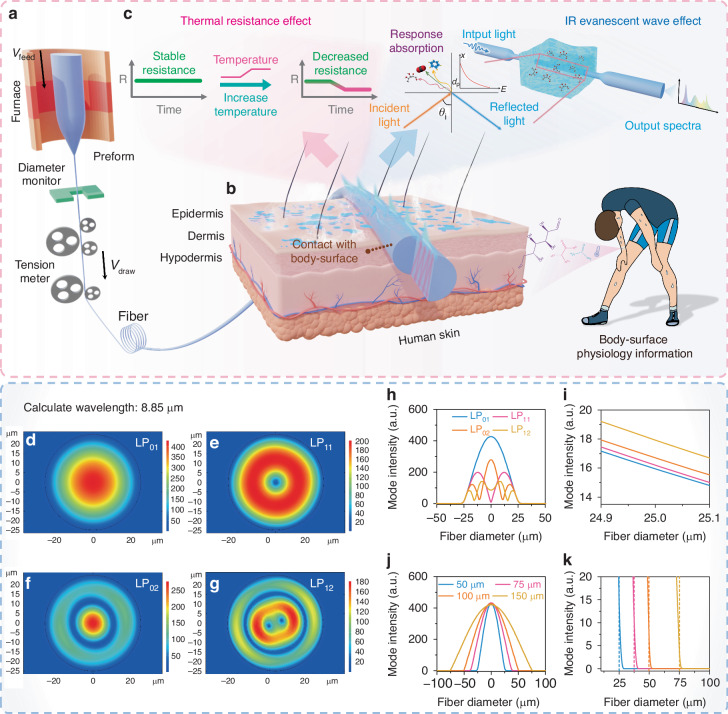
The concept of AST fiber-based body-surface monitoring technique. **a** Schematic of the fiber-drawing process in the thermal fiber-drawing tower. **b** Skin as a diagnostic platform showing diagnostic signals from temperature and sweat. **c** Principle of thermal resistance and fiber evanescent wave effect for temperature and IR sensing. **d–g** Mode simulation and field intensity distribution of AST fibers with different geometries. **h** Field distribution of different modes. **i** Boundary intensity details of different modes, increasing with the mode order. **j** Field intensity plot of fibers with different diameters. **k** Boundary intensity details of fibers with different diameters

Figure 1a schematically depicts the fabrication process of the ChG fibers. Initially, a semiconducting glass rod of the AST was synthesized using standard sealed ampoule technique. This semiconducting glass exhibits two significant properties for IR-temperature sensing. Firstly, its resistance decreases significantly with increasing temperature, displaying a pronounced negative temperature coefficient behavior^38,39^. Secondly, it exhibits high resistance to crystallization and a wide IR transmission range (Fig. S1), making it particularly suitable for the thermal fiber drawing process and for application in MIR-FEW. The preform was heated in the furnace until the bottom melted to a viscous state, and then it was pulled down by gravity. The fiber diameter could be controlled from micrometers to millimeters by adjusting the feeding and drawing speeds. As shown in Fig. S2, the amorphous structure of the AST glass was maintained after the fiber drawing process. Figure S3 shows the fiber losses in 2.5-13 μm range, with a minimum loss of 3.3 dB m^–1^ at 9.2 μm, and no significant impurity absorption between 5.5 and 12.5 μm, which is beneficial for the detection of substances. The fiber with a diameter of 450 μm demonstrated good roundness (Fig. S4a), and the EDS mapping confirmed that As, Se, and Te elements were homogeneously distributed within the fiber (Fig. S4b–d). The EDS profile of the AST fiber (Fig. S4e) indicated that the atomic ratio of As:Se:Te in the fiber was approximately 29:50:21, which was consistent with the initial composition of the precursor glass.

The mechanism of IR-temperature sensing using an AST fiber is shown in Fig. 1b, c. As shown in Fig. 1b, sweat glands and pores are distributed widely throughout the skin surface and play a crucial role in regulating core body temperature and excreting metabolic products through sweating^40^. Disruptions in this heat-dissipation process can impair bodily functions due to high heat loads, which are common in diseases such as fever and hyperthyroidism^41^. Therefore, monitoring sweat biomarkers and temperature is essential for comprehensive understanding of health conditions and accurate disease diagnosis. The AST fiber, due to its thermal resistance effect, varies in resistance when the body temperature fluctuates, allowing for effective skin temperature monitoring. Additionally, sweat biomarkers can be qualitatively analyzed based on their unique spectral fingerprints in the MIR-FEWS absorbance spectra after the AST fibers come into contact with sweat (Fig. 1c).

When a multimode AST fiber is used for MIR-FEW sensing, hundreds of modes can propagate through the fiber^42^. COMSOL Multiphysics was employed to simulate the propagation modes in fibers with different geometries through finite element analysis, providing insight into the intensity distribution of evanescent waves. Figure 1d shows the mode field distribution of the LP_01_ mode for a fiber with a diameter (*d*_w_) of 50 μm. The refractive index of the AST fiber was 2.92 at 8.85 μm, while the refractive index of the external medium was set to 1.33 to simulate an aqueous solution. As the fiber mode order increased, the mode field distribution became more complex (Fig. 1e–g), Fig. 1h displays the corresponding 1D plots. As shown in Fig. 1i, the light intensity decayed exponentially into the cladding medium near the fiber boundary. Compared to the fundamental mode, higher-order modes penetrate further into the cladding medium, thereby enhancing the measurement sensitivity. This is the main advantage of using multimode AST fibers for MIR-FEW sensing. Additionally, the MIR-FEW sensitivity of the AST fiber is influenced by both the penetration depth (${d}_{\text{p}}$) and the total number of light reflections (*N*), which can be quantified using the following formulas^29,36^:

$${{d}}_{\text{p}}=\frac{\lambda }{2\pi\sqrt{{n}_{1}^{2}{\sin }^{2}{\theta }_{{\rm{i}}}-{n}_{2}^{2}}}$$

$$N={l}_{w}\times \frac{\tan (90-{\theta }_{\text{i}})}{{d}_{w}}$$

where $\lambda$, ${\theta }_{\text{i}}$ and ${l}_{\text{w}}$ are the wavelength, incident angle of the light, and fiber length in the sensing area, respectively. *n*_1_ and *n*_2_ are the refractive indices of the core and external medium, respectively. A decrease in fiber diameter results in a smaller ${\theta }_{\text{i}}$, which leads to an increase in ${d}_{\text{p}}$. Additionally, increasing the sensing fiber length enhanced the interaction between the FEW and the substance. Therefore, reducing the fiber diameter and increasing the sensing fiber length are effective methods for enhancing the FEW intensity of AST fibers. Figure 1j shows the variation in the intensity distribution in the LP_01_ mode for different fiber diameters. As shown in Fig. 1k, the mode field intensity at the boundary increased gradually as the fiber diameter decreased.

### MIR-FEW sensing performance of the AST fiber

**Fig. 2:**
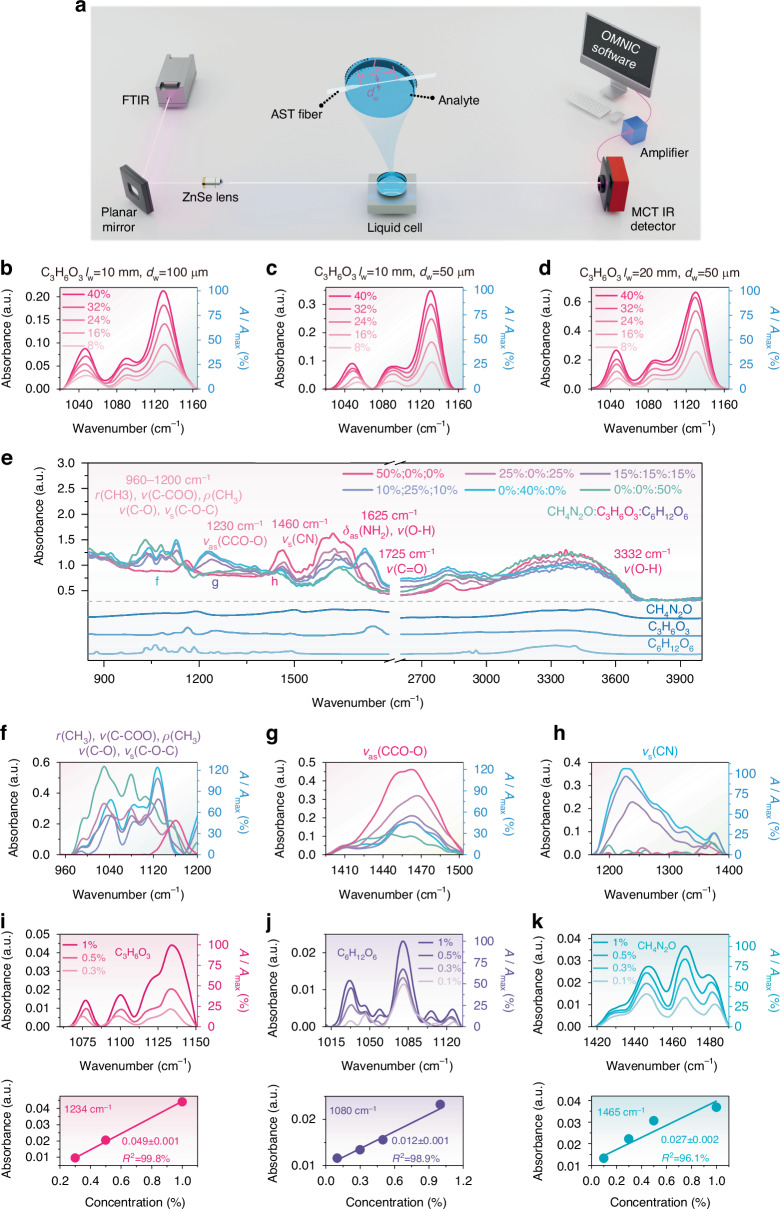
Spectral response of AST fiber to sweat biomarkers. **a** Schematic of the IR sensing platform. Absorption spectra and relative absorbance (*A*/*A*_max_, where *A* represents absorbance, and *A*_max_ represents the maximum absorbance) of C_3_H_6_O_3_ with different concentrations detected by AST fibers with different sizes: **b** *l*_w_ = 10 mm, *d*_w_ = 100 μm, **c** *l*_w_ = 10 mm, *d*_w_ = 50 μm, **d** *l*_w_ = 20 mm, *d*_w_ = 50 μm. **e** Evanescent wave absorption spectra of mixtures of CH_4_N_2_O, C_3_H_6_O_3_, and C_6_H_12_O_6_ with different proportions. **f** *v*(C-O), *v*_s_(C-O-C), *r*(CH_3_), *v*(C-COO) and *ρ*(CH_3_) absorption bands in the mixtures. **g** *v*_as_(CCO-O) absorption bands in the mixtures. **h** *v*_s_(CN) absorption band in the mixtures. Sensing results of low-concentration (**i**) C_3_H_6_O_3_, (**j**) C_6_H_12_O_6_, and (**k**) CH_4_N_2_O solutions

To evaluate the sensing performance of the MIR-FEWS, we set up an experimental apparatus (Fig. 2a) that included a FTIR spectrometer, off-axis parabolic gold mirror, ZnSe objective lens, liquid cell, mercury cadmium telluride (MCT) detector, and computer. The formulas and simulation results indicated that optimizing the structure of the fiber can enhance its sensing performance, specifically by reducing the fiber diameter and increasing the sensing fiber length. Consequently, we created three fiber structures using the biconical taper method to enhance the FEW intensity. The parameters of the sensors are as follows: I: *l*_w_ = 10 mm, *d*_w_ = 100 μm, II: *l*_w_ = 10 mm, *d*_w_ = 50 μm, III: *l*_w_ = 20 mm, *d*_w_ = 50 μm. Figure 2b–d shows the sensing results of the three AST fibers when detecting various concentrations of lactic acid (C_3_H_6_O_3_) solution. This detection was based on monitoring the spectral fingerprint of C-O-C stretching vibration at 1130 cm^−1^, which is used to evaluate its sensing performance^43^. As the C_3_H_6_O_3_ concentration increased, the intensity of the characteristic peaks showed a significant enhancement. At the same C_3_H_6_O_3_ concentration, the absorbance intensity increased with a decrease in *d*_w_ and an increase in *l*_w_. Notably, the AST fiber with *l*_w_ = 20 mm and *d*_w_ = 50 μm achieved a sensitivity of 0.0104 a.u. %^–1^ (Fig. S5), and the *d*_p_ of evanescent wave of AST fiber was also evaluated and shown in Fig. S6.

To evaluate the capability of the AST fiber to detect sweat components, we focused on glucose (C_6_H_12_O_6_), urea (CH_4_N_2_O), and lactic acid (C_3_H_6_O_3_), which are common sweat biomarkers related to diseases such as hyperthyroidism, diabetes, and chronic kidney disease^44^. As shown in Fig. 2d, we have demonstrated the ability of the AST fiber to detect C_3_H_6_O_3_. Owing to the wide MIR transmission range and high sensitivity of the AST fibers, they can also be used to detect C_6_H_12_O_6_ and CH_4_N_2_O (Fig. S7). Furthermore, the accuracy of the AST fibers was evaluated using 10 tests with 5% C_6_H_12_O_6_, 5% C_3_H_6_O_3_ and 5% CH_4_N_2_O. As shown in Fig. S8, the average detection error of the AST fibers was within 2%, indicating high accuracy. The capability of simultaneous multicomponent detection is crucial for analyzing the complex composition of human sweat. Therefore, different concentrations of mixed solutions containing CH_4_N_2_O, C_3_H_6_O_3_ and C_6_H_12_O_6_ were prepared for testing. As shown in Fig. 2e, all the peaks were identified and matched well with the characteristic vibration peaks of CH_4_N_2_O, C_3_H_6_O_3_ and C_6_H_12_O_6_ (assignments are summarized in Table S1 and Fig. S9). The C_6_H_12_O_6_ solution exhibited characteristic absorption peaks attributed to the *v*(C-O) and *v*_s_(C-O-C) at 960–1200 cm^–1^ (Fig. 2f). With an increase in the concentration of CH_4_N_2_O, the changes in the characteristic absorption peak pattern of C_6_H_12_O_6_ at 960–1200 cm^–1^ were negligible. Subsequently, as the concentration of C_3_H_6_O_3_ in the mixed solution was further increased, the overlapping absorption bands of C_6_H_12_O_6_ at 1030 cm^–1^ and C_3_H_6_O_3_ at 1042 cm^–1^ broadened the characteristic absorption peak of the mixed solution. Furthermore, the strong absorption peak of C_3_H_6_O_3_ at 1130 cm^–1^ was observed in the mixed solution. However, owing to the excessive overlap of the characteristic peaks of C_6_H_12_O_6_ and C_3_H_6_O_3_, quantitative analysis was difficult. Fortunately, C_3_H_6_O_3_ exhibits a unique *v*_as_(CCO-O) at 1230 cm^−1^, which was very weak in both C_6_H_12_O_6_ and CH_4_N_2_O (Fig. 2g)^45^. Consequently, it was selected for the quantitative analysis of C_3_H_6_O_3_. As per the intensity of *v*_as_(CCO-O) at 1230 cm^–1^, the absorption peak intensity at 1030 cm^−1^ can be determined, allowing for the quantitative analysis of C_6_H_12_O_6_. Additionally, CH_4_N_2_O displayed a *v*_s_(CN) band at 1460 cm^−1^ (Fig. 2h)^46^, which was selected for the quantitative analysis of CH_4_N_2_O. For example, a mixed solution containing 10% CH_4_N_2_O, 10% C_3_H_6_O_3_, and 10% C_6_H_12_O_6_ was tested. By calibrating the intensities of the characteristic peaks of each substance, the content of each substance in the mixed solutions was successfully determined (Fig. S10). Due to the low concentrations of CH_4_N_2_O, C_3_H_6_O_3_ and C_6_H_12_O_6_ in sweat, it is crucial to evaluate the detection capabilities of the AST fibers for trace amounts of these substances. The characteristic peaks of C_3_H_6_O_3_, C_6_H_12_O_6_ and CH_4_N_2_O weakened with decreasing concentrations, with detection limits of 0.3%, 0.1%, and 0.1%, respectively (Fig. 2i–k). It is noteworthy that the detection sensitivity of AST fibers significantly increases at low concentrations compared to high concentrations. This increase is attributed to the hydrophobicity of the AST glass (Fig. S11), which is consistent with previous reports^47,48^.

### Temperature-sensing performance of the AST fiber

**Fig. 3:**
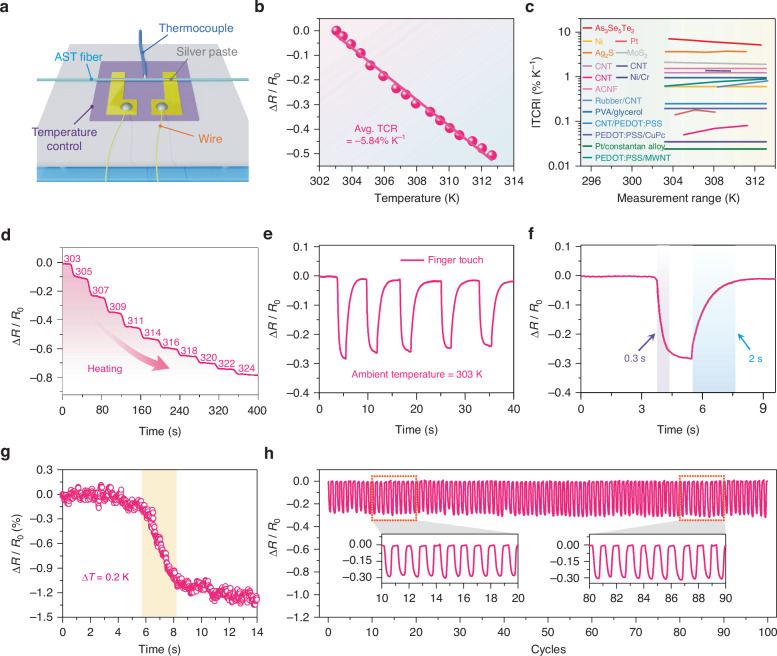
Electrical response of AST fiber to temperature stimulation. **a** Schematic of AST fiber resistance temperature variation testing platform. **b** Resistance-temperature curves for an AST fiber. **c** Comparison of absolute value of the temperature coefficient of resistance |TCR| of recently reported temperature sensors in the human body temperature range of 303–313 K. **d** Stable resistive response at different dynamic temperature gradients. **e** Relative resistance variation of the AST fiber for loading and unloading actions by the touch of a finger. **f** Response-recovery time after finger touch. **g** Relative resistance variation of the AST fiber, resulting in a minimum discernible temperature difference of 0.2 K. **h** Relative resistance variation of the AST fiber under 100 cycles of finger-touch stimulation

To investigate the temperature-sensing properties of the AST fiber, an experimental apparatus was established consisting of a temperature-control platform, thermocouple, and AST fiber (Fig. 3a). The fiber resistance change rate (Δ*R*/*R*_0_) was approximately 50% from 303 to 313 K, indicating its high suitability for skin temperature sensing (Fig. 3b). Additionally, by fitting the normalized resistance relative to 303 K, the TCR was calculated as –5.84% K^–1^ within the 303–313 K range, higher than all other temperature sensing materials (Fig. 3c). Figure 3d illustrates the resistance variation of the AST fiber with temperature fluctuations in the range of 303 to 324 K. The resistance decreased in a stepwise manner with increased temperature, and at each incremental applied temperature, the resistance changed smoothly and rapidly. To verify the sensing performance in practical applications, we tested the response of the fibers to finger touches at room temperature (303 K). As shown in Fig. 3e, the AST fibers exhibited excellent response repeatability. Notably, the AST fiber displayed a fast response-recovery time of 0.3 s and 2 s, respectively (Fig. 3f). Furthermore, AST fiber was able to sense temperature differences as small as Δ*T* = 0.2 K (Fig. 3g), which is crucial for detecting subtle temperature variations. In addition, the AST fiber exhibited a stable cyclic response for temperature sensing under finger temperature simulation (Fig. 2h).

### Dual-mode sensing applications of the AST fiber

Before conducting operando measurements in sweat biomarker and temperature sensing using AST fibers, several considerations regarding the reliability of the measurements need to be addressed. First, the refractive index of ChGs varies with the temperature, which affects the penetration depth of the FEW and, consequently, the absorbance^26^. Therefore, it is crucial to assess whether temperature affects the results, especially considering potential temperature fluctuations during disease progression. To account for the possibility of evaporation affecting the aqueous solution concentration during the heating process, we conducted tests using silicone oil (C_2_H_8_O_2_Si) and dichloromethane (CH_2_Cl_2_). With a temperature change of 2 °C, the measured change in signal absorbance fell within the margin of error (Fig. S12). Additionally, we measured the refractive index changes of AST glass within the range of 30–42 °C. The refractive index changed by less than 0.1% as the temperature increased from 30 to 42 °C (Fig. S13). Using the refractive index at 30 °C and 42 °C, we simulated the evanescent field intensity and found that the intensity remained virtually unchanged. Hence, any temperature effect can be safely disregarded when using FEW to detect sweat biomarker within the temperature range of the human body. Secondly, the AST fibers were immersed in simulated sweat at 40 °C for 24 h, cycled 10 times to evaluate their long-term durability. The SEM results show no observable changes on the fiber surface (Figs. S14, 15). Altogether, these results indicate that the sweat and temperature-sensing capabilities of the AST fibers can operate independently and without interaction with the skin environment.

**Fig. 4:**
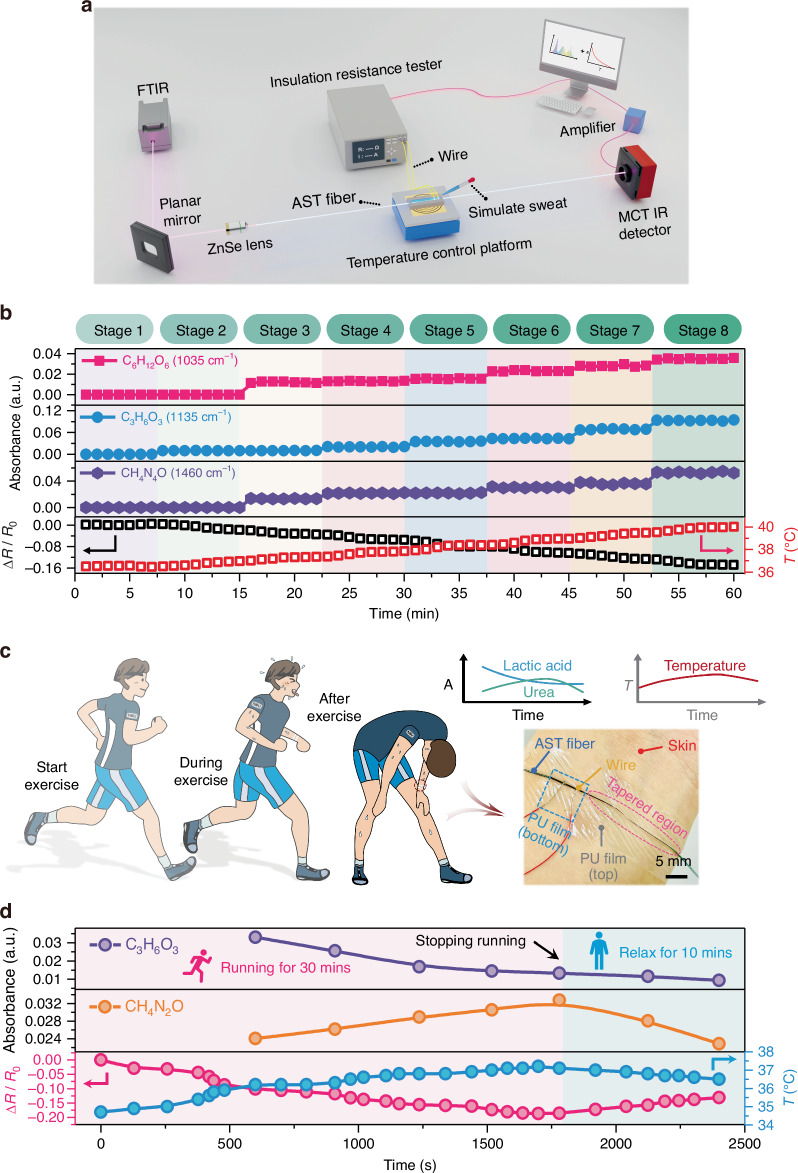
Real-time monitoring of sweat biomarkers and temperature. **a** Schematic of the dual-sensing platform for sweat biomarker and temperature measurements using an AST fiber. **b** Sensing ability of the AST fiber when the sweat biomarker concentration and temperature change simultaneously. **c** Schematic and photograph of sweat-temperature monitoring during exercise. **d** Real-time, continuous, in situ measurement of the temperature and sweat (C_3_H_6_O_3_ and CH_4_N_2_O) levels from the wrist of a healthy subject during exercise, which includes 30 min of running and 10 min of rest

After these tests, we investigated the body-surface physiological sensing ability of the AST fibers. Figure 4a shows the experimental setup for conducting sweat biomarker and temperature sensing using the AST fiber. This setup includes a FTIR spectrometer, a MCT detector, a temperature control platform, a resistance testing device, and a segment of AST fiber (the actual test setup is shown in Fig. S16). The resistance and absorbance intensity of the AST fiber under different temperatures and biomarker concentrations are shown in Fig. 4b. Initially, the fiber is maintained at 36.5 °C without any biomarkers. In subsequent stages, the temperature is increased in 0.5 °C increments, accompanied by a gradual rise in the biomarker concentrations (Table S3). The results show that as the concentrations of C_6_H_12_O_6_, C_3_H_6_O_3_, and CH_4_N_2_O increase, the intensities of their respective characteristic peaks also increase (Movie S1 and S2). Notably, while the resistance of the AST fiber decreases progressively with increasing temperature, the absorbance intensity of the characteristic peaks remains largely unchanged within the same stage. These results further indicate that temperature variations do not compromise the ability of the AST fiber to detect sweat biomarkers. Its dual-mode sensing properties and independent sweat biomarkers and temperature detection capabilities make it a promising candidate for disease progression monitoring and prevention.

To further demonstrate the practical application of the AST fiber, it is coated with a PU film and then attached to the wrist of a healthy subject to monitor the variations in sweat CH_4_N_2_O, C_3_H_6_O_3_, and body temperature during exercise (Fig. 4c and S17). Here, the PU film with excellent flexibility and elasticity can serve as a cushioning layer to withstand external pressure and stress applied to the fiber taper region and prevent the fiber from breaking. Figure 4d illustrates the corresponding real-time measurements on the subject’s wrist during exercise with the AST fiber (exercise phase: 0–1800 s, relaxation phase: 1800–2400 s, and the subject started to sweat after approximately 600 s of exercise), and the test process is shown in Fig. S18. The resistance of the AST fiber continuously decreased during the 30 min of running, with the wrist temperature increasing from 34.7 to 37.2 °C. After stopping the exercise, the body temperature decreased from 37.2 °C to 36.5 °C within 10 min due to increased sweating and evaporative cooling. Additionally, we observed a decreasing trend in sweat C_3_H_6_O_3_ concentration. This is owing to the rapid anaerobic glycolysis during the initial 10 minutes of exercise, which caused the C_3_H_6_O_3_ concentration to rise to a high level. Afterward, as the body transitions to aerobic metabolism (from 10 min), C_3_H_6_O_3_ production diminishes, leading to a gradual reduction in its concentration. This phenomenon is also referred to as the lactic acid threshold in physiology^49,50^. In contrast, the concentration of CH_4_N_2_O in sweat gradually increases with exercise intensity, reflecting the acceleration of protein metabolism in the body. During this process, protein is broken down into CH_4_N_2_O and excreted through sweat. After exercise ends, the rate of protein breakdown slows down, and metabolic demands decreases, leading to a reduction in CH_4_N_2_O production and a gradual decline in its concentration during rest. These results indicate that AST fibers with biomarker- and temperature-sensing capabilities have great potential for personalized physiological and metabolic monitoring.

## Discussion

In summary, a simple multifunctional AST fiber was designed and fabricated to monitor sweat biomarkers and body temperature simultaneously. The incorporation of thermal resistance and fiber evanescent wave effects enabled the simultaneous detection of sweat biomarkers and temperature without the need for an additional decoupling process, representing an advancement in the field of body-surface monitoring. With the high |TCR| (5.84% K^−1^) of the AST fiber and enhanced intensity of FEW after tapering, the fiber achieved high sensitivity in IR and temperature sensing. Importantly, the continuous detection capability of the AST fiber for temperature and sweat biomarkers during exercise demonstrates its applicability in a wide range of personalized diagnostic and physiological monitoring applications.

## Materials and methods

### Fabrication of the AST fiber

The AST glass samples were synthesized by the melt-quenching technique. High-purity elements, including As, Se, Te (99.999%), and 0.3% Mg, were accurately weighed in an Ar-protected glove box and placed in a distillation tube, which was connected to a turbo-molecular pump system. The raw materials were heated at 200 °C for 1 h to remove absorbed water and volatile impurities while pumping. Subsequently, the distillation tube was sealed with a torch when the vacuum reached 10^−5 ^Pa. Thereafter, the distillation tube was gradually heated from room temperature to 900 °C. During heating, all elements except Mg were distilled to the low-hydroxyl silica ampoule, while oxygen reacted with Mg, and other impurities eliminated in the distillation tube. The low-hydroxyl ampoule tube was then separated from the distillation with a torch. The distilled mixture in the sealed ampoule was held at 850 °C for 12 h and quenched in water to form a glass rod. After annealing around the glass transition temperature (*T*_g_), a preform with a diameter of 15 mm and a length of 80 mm was obtained. To mitigate the influence of preform surface defects on the optical and mechanical properties of the fiber, the glass preform was polished. Fibers were fabricated from the polished preforms under a He atmosphere using a custom-made fiber tower. During the drawing step, the diameters of the fibers were controlled to be in the range of 400–450 μm by adjusting the preform-feeding and fiber-drawing speed.

### Preparation of tapered AST fiber

Two Thorlabs NRT150/M high-precision steppers equipped with fiber holders were used to fix the fiber with a total length of 50 cm. An electric heating element positioned at the center of the fiber raised the temperature to 170–200 °C. At this critical temperature range, the fiber began to undergo slight deformation due to gravitational forces, initiating the tapering process. A digital microscope was used to monitor the taper drawing process of the AST fiber in real time. The tapering process was initiated upon observing the initial deformation of the fiber. The motion program and parameters, including the direction, speed, distance, and dwell time of the steppers, can be set in advance independently.

### Material characterization and testing

A 2 mm-thick glass disk cut from the glass preform was polished on both sides and tested using an FTIR spectrometer (Nicolet 380) to measure its IR transmission spectra. The thermal behaviors were investigated using differential scanning calorimetry (DSC, Q2000, TA). The structural analyses of the glass and fiber were conducted using X-ray diffraction (XRD, D2 phaser, Bruker), and further investigations were conducted using a micro-Raman spectrometer (Renishaw inVia, London, UK) with 785 nm laser excitation. The fiber loss was measured using the cut-back method. Specifically, 1 m of fiber was sequentially cut back to 0.8 m, 0.6 m, 0.4 m, and 0.2 m, enabling the acquisition of four loss spectra by comparing the IR transmission spectra through the original length of the fiber to each shortened length. Subsequently, an average loss spectrum was calculated and plotted. The MIR-FEWS sensing performance was evaluated using an optical platform equipped with a FTIR spectrometer. Due to the large beam size of the FTIR apparatus, an off-axis parabolic gold mirror was used for focusing. The light was further focused by a ZnSe MIR focusing objective lens. The liquid cell and mercury cadmium telluride (MCT) detector were positioned on three-dimensional translation stages to ensure optimal coupling efficiency. The outgoing signal was detected by an external MCT detector and analyzed by OMNIC software to obtain the evanescent wave spectra. The MIR absorption spectrum of the liquid was collected through the tapered fiber 10 times. The temperature-sensing performance under different temperatures was measured by an insulation resistance tester (TH2683).

## Supplementary information

Supplementary information

Movie S1

Movie S2

## Data Availability

All data are available in the main text or the supplementary materials. Information requests should be directed to the corresponding authors.
