# Supplementary material for: Unlocking body-surface physiological evolution via IR-temperature dual sensing with single chalcogenide fiber
Source: Light Sci Appl. 2025 Apr 25;14:173. doi: 10.1038/s41377-025-01840-y (PMC12032074; doi:10.1038/s41377-025-01840-y)
Supplement: Supplementary file 1 — Supplementary information [file 41377_2025_1840_MOESM1_ESM.docx]

Supplementary Information for

**Unlocking Body-Surface Physiological Evolution Via IR-Temperature Dual Sensing with Single Chalcogenide Fiber**

Yanqing Fu,^1,2,3^ Shiliang Kang,^1,2,3*^ Gangjie Zhou,^1,2,3^ Xinxiang Huang,^1,2,3^ Linling Tan,^1,2,3^ Chengwei Gao,^1,2,3^ Shixun Dai,^1,2,3^ Changgui Lin^1,2,3*^

*^1^* *Laboratory of Infrared Materials and Devices,* *The Research Institute of Advanced Technologies, Ningbo University, Ningbo 315211, China*

*^2^ Zhejiang Key Laboratory of Advanced Optical Functional Materials and Devices, Ningbo 315211, China*

*^3^ Engineering Research Center for Advanced Infrared Photoelectric Materials and Devices of Zhejiang Province, Ningbo 315211, China*

***Corresponding author:** kangshiliang@nbu.edu.cn; linchanggui@nbu.edu.cn

**This supplement includes:**

Figure S1 to S18

Table S1 to S3

Legends for movies S1 and S2

References


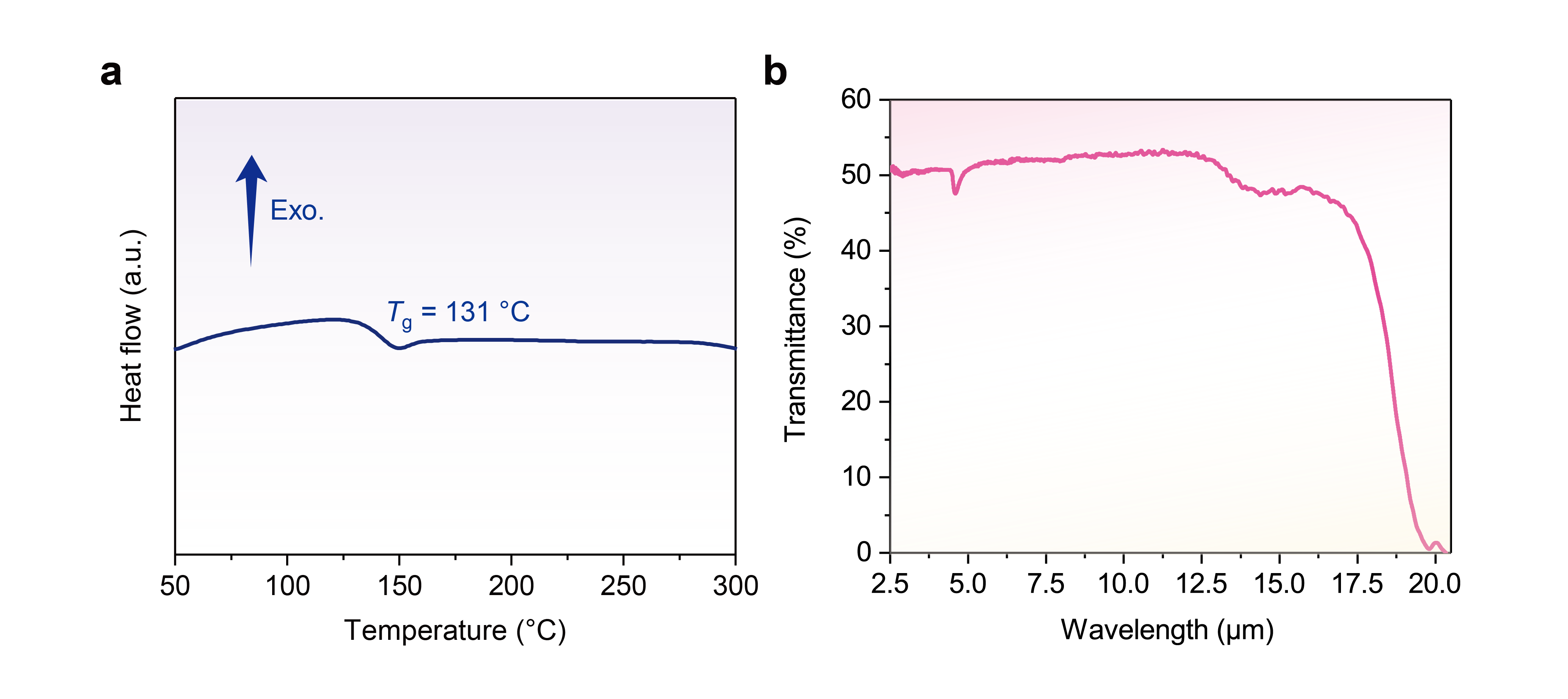


**Figure S1 a** The DSC curve of AST glass. **b** The transmission spectrum of AST glass. It can be seen the *T*_g_ of the AST glass is 131 °C. When the temperature rises to 300 °C, there is still no crystallization peak, indicating that this glass has good thermal stability and can be used to further thermal drawing and tapering. Furthermore, the AST glass shows good transmittance over 2.5-15 μm (approximately 50%), and there is no obvious absorption except for Se-H bond at 4.5 μm.


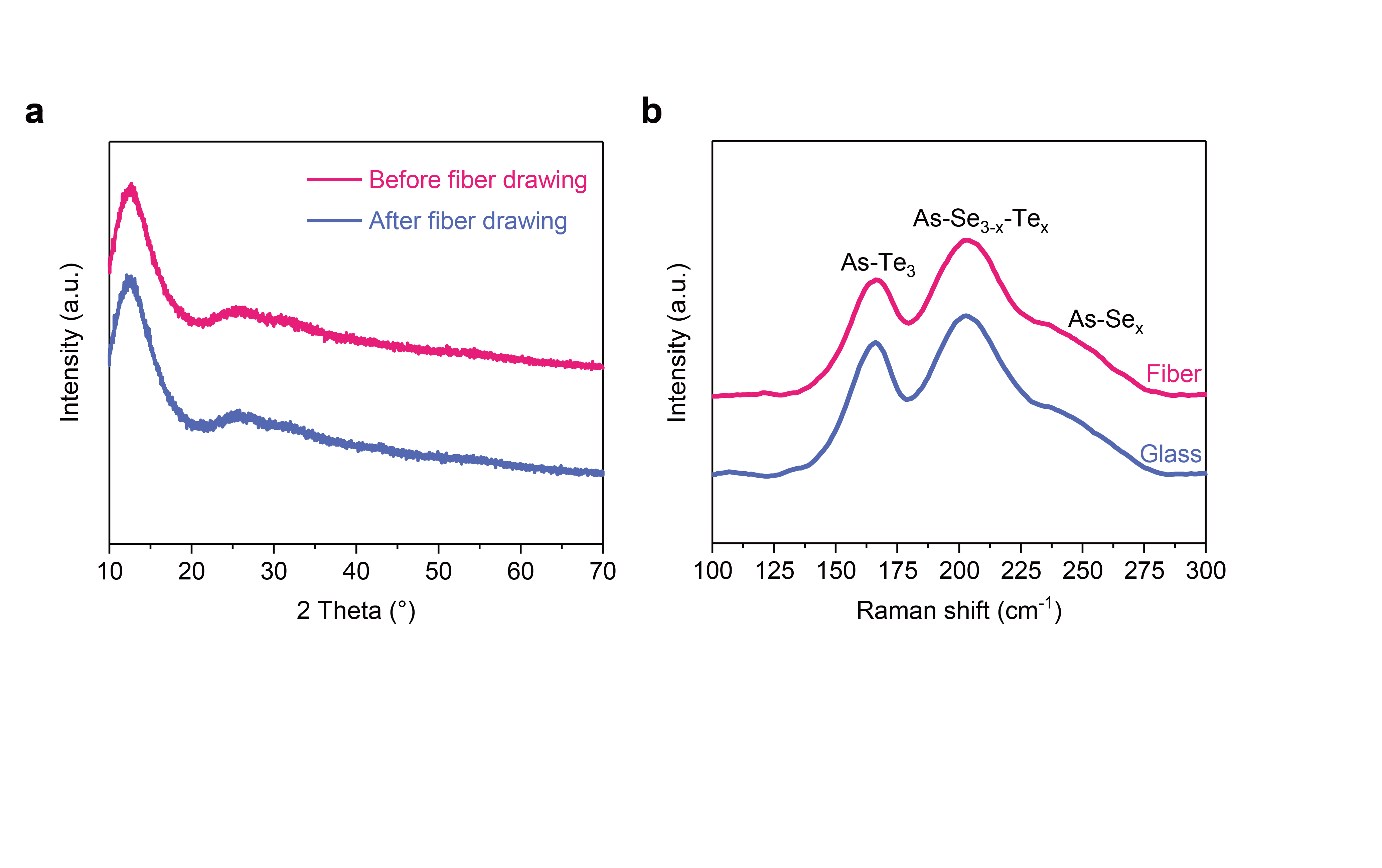


**Figure S2 a** XRD patterns of AST glass and fiber. **b** Micro-Raman spectra of AST glass and fiber. The broad peaks in the glass and fiber are indicative of the amorphous nature, implying that the amorphous structure of AST glass can be well maintained during the fiber drawing process. The measured Raman spectra of AST glass and fiber exhibit three distinct features at 163, 205 and 250 cm^-1^. The main band near 205 cm^-1^ are characteristics of stretching and bending modes of As-Se_3-x_Te_x_, and two shoulder at the lower (163 cm^-1^) and higher (250 cm^-1^) frequency are attributed to As-Te_3_ and As-Se_x_ bonds, respectively. There is no significant change in the peak pattern between AST glass and fiber, which also indicated the structure of AST glass was well maintained after fiber drawing.


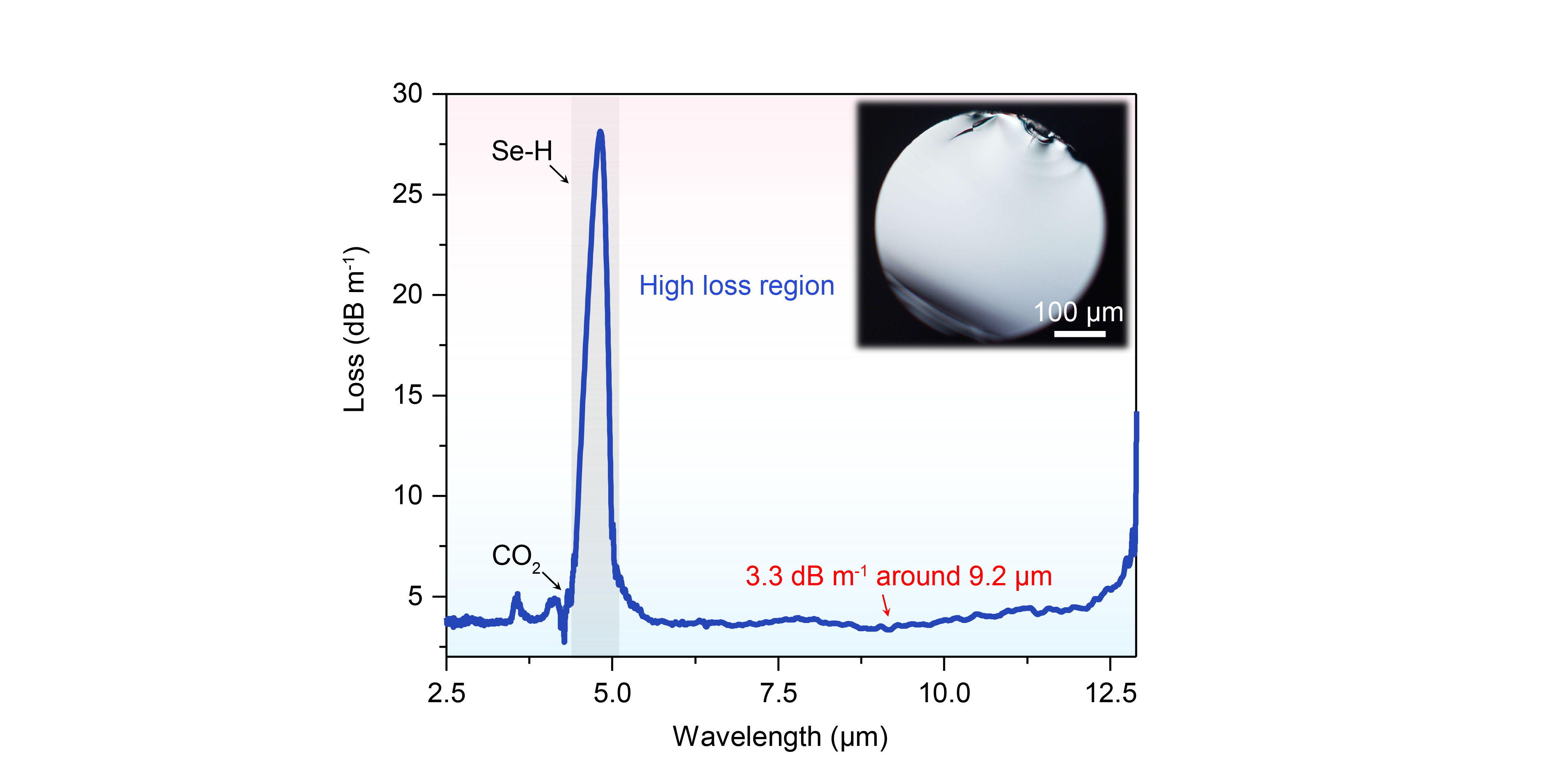


**Figure S3** Loss spectrum of the AST fiber tested by the cut-back method. The inset is the end face of the fiber. The Figure S3 shows the loss spectrum, where a transmission window from 2.5 to 13 μm with the loss minimum of 3.3 dB m^-1^ at 9.2 μm. Moreover, the overall loss was lower than 5 dB m^-1^ between 2.5 to 12 μm. In addition, the loss spectrum also presents some impurity absorptions, for instance, the CO/CO_2_ at 4.4 μm and Se-H at 4.6 μm, which are consistent to the absorption spectrum of the AST glass.


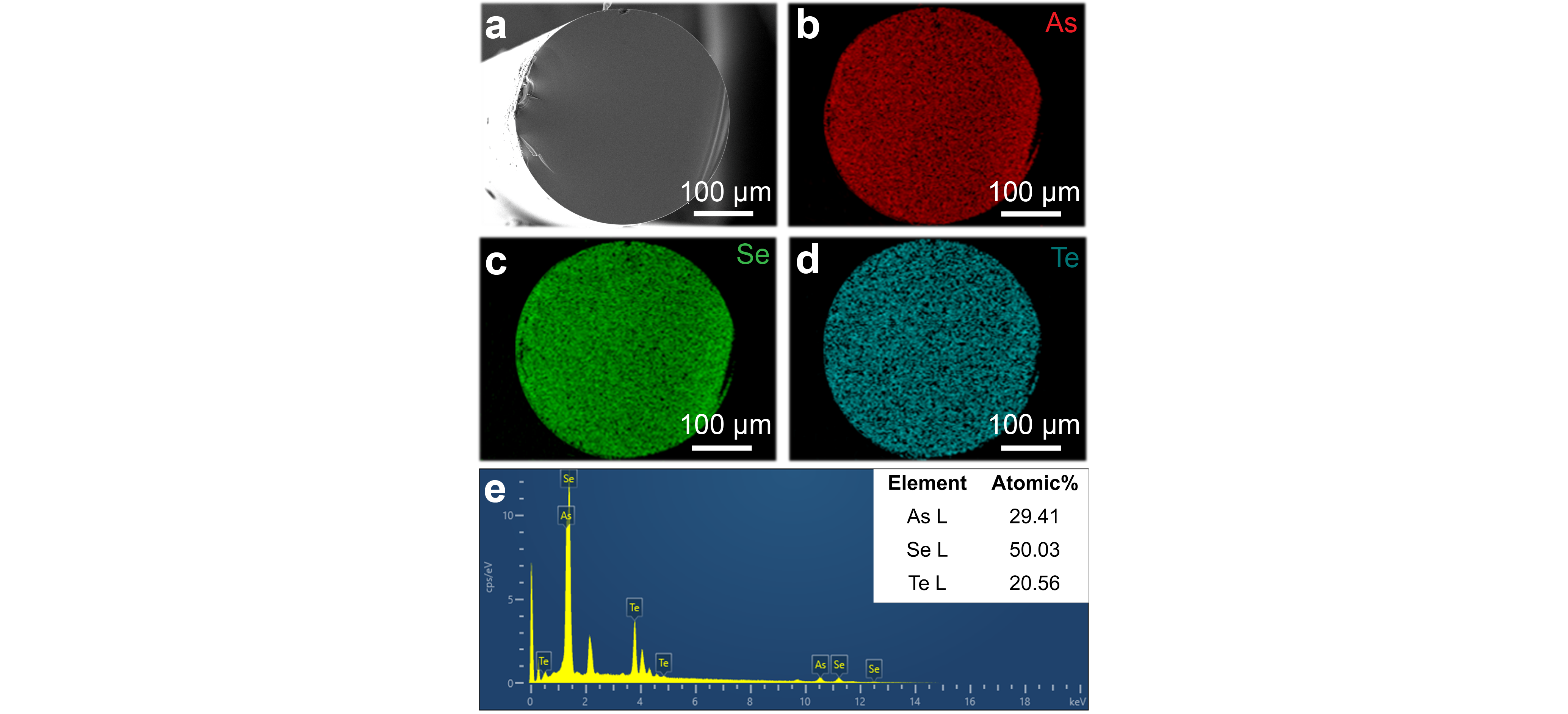


**Figure S4** SEM-EDS results of the fiber and face. **a** The SEM of the fiber cross-section. **b-d** The SEM mapping of As, Se and Te elements in the AST fiber. **e** The EDS spectra of the AST fiber. The results show the fiber diameter is 450 μm, and the elements are uniformly distributed in the fiber. Furthermore, the ratio of As:Se:Te is about 29:50:21, which is close to the original composition of AST glass.


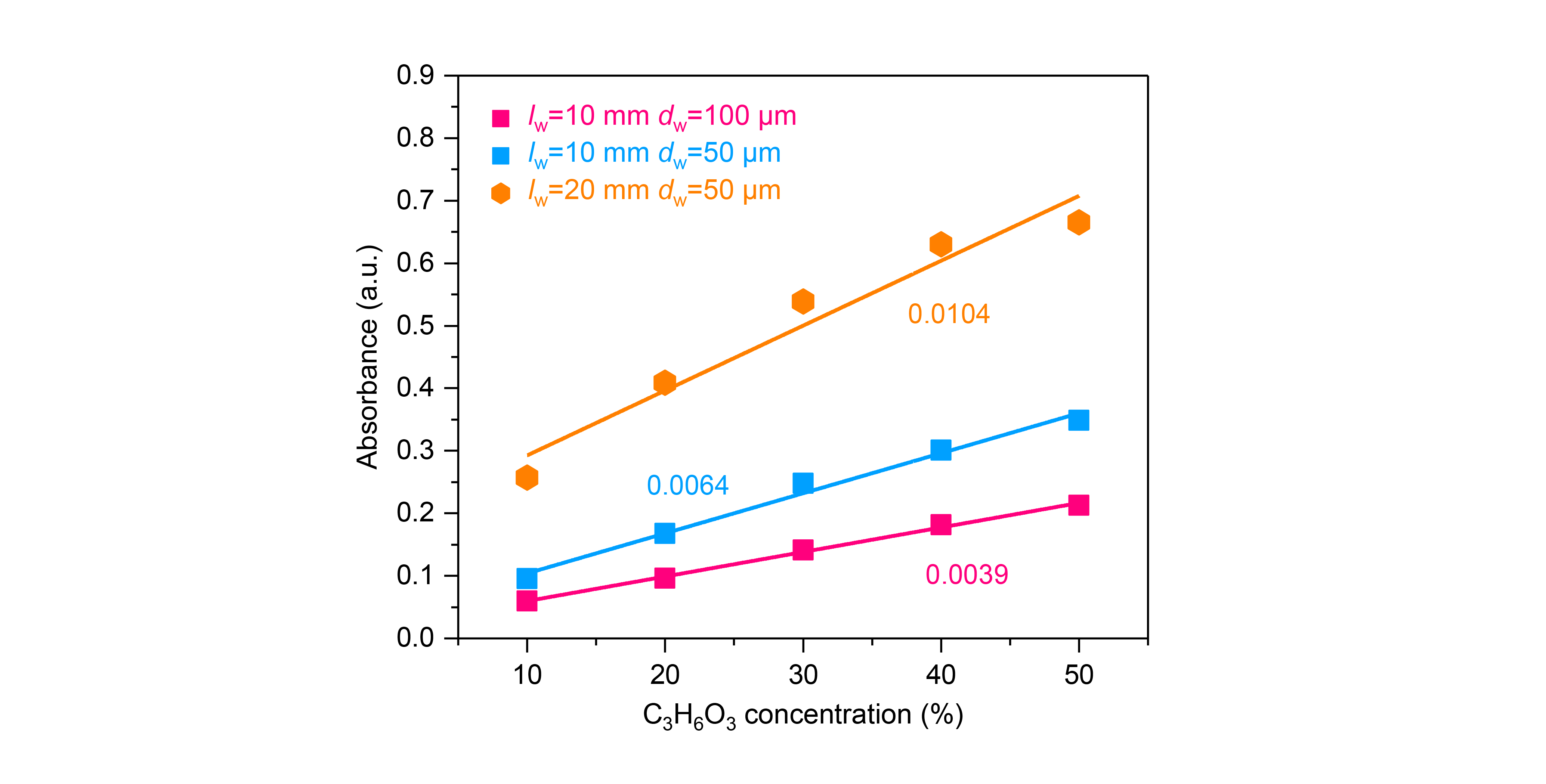


**Figure S5** Sensitivities of AST fibers with different *l*_w_ and *d*_w_. For the detection of C_3_H_6_O_3_, the sensitivity of the AST tapered fiber with *l*_w_=20 mm and *d*_w_=50 μm can reach 0.0104 a.u. %^-1^. The fiber can be further drawn to smaller dimensions, however, it is difficult for post-processing due to poor mechanical strength. Hence, the fiber with *l*_w_=20 mm and *d*_w_=50 μm was chosen for FEW sensing.


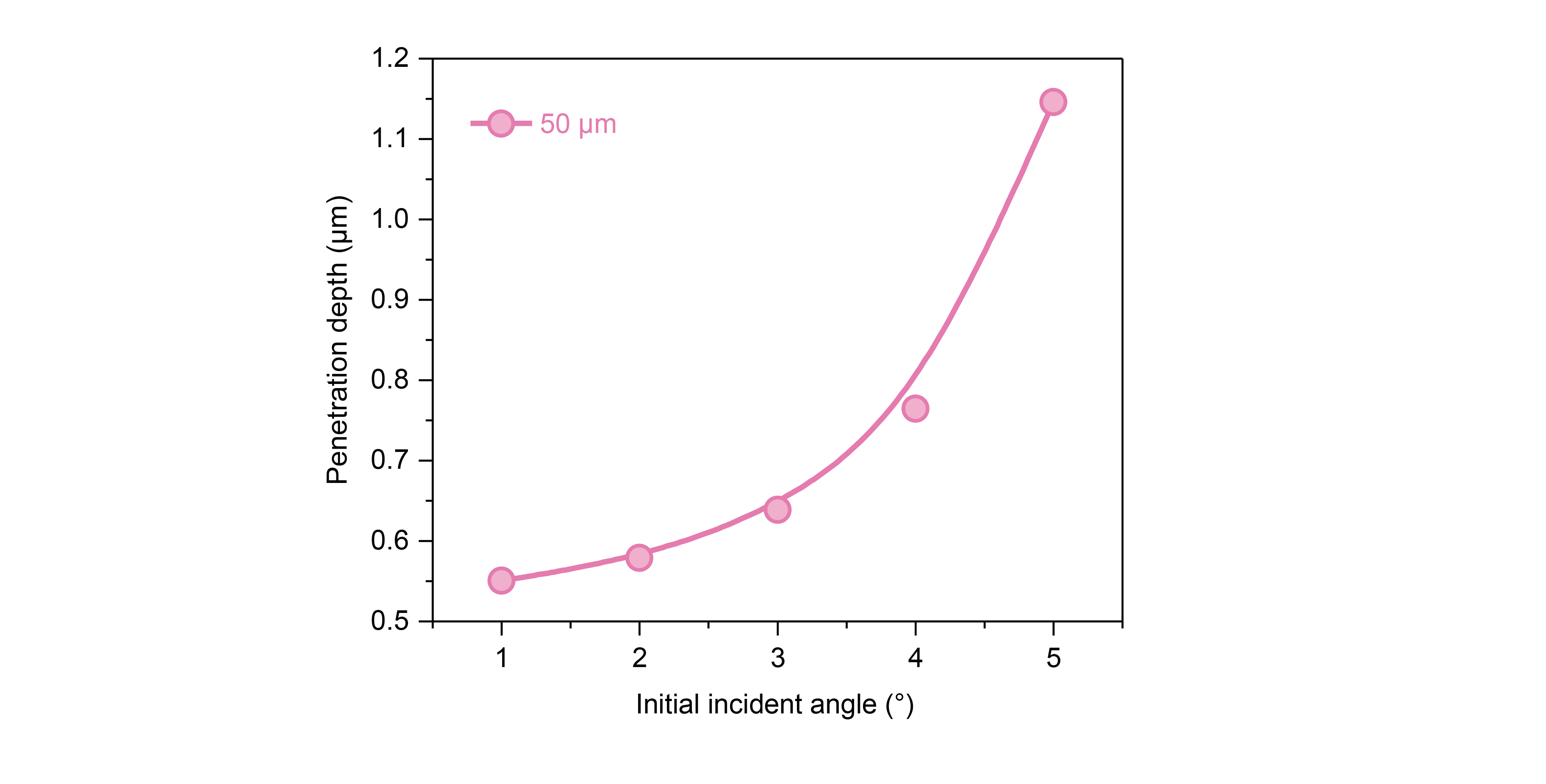


**Figure. S6** The evanescent wave penetration depth $\text{d}_{\text{p}}$ of tapered AST fiber with diameter of 50 μm at varying initial incident angles. The initial incident angle (*α*) has a significant impact on the $\text{d}_{\text{p}}$. With the increase of *α* from 1 to 5°, the $\text{d}_{\text{p}}$ of the AST tapered fiber enhances from 0.55 to 1.14 μm.


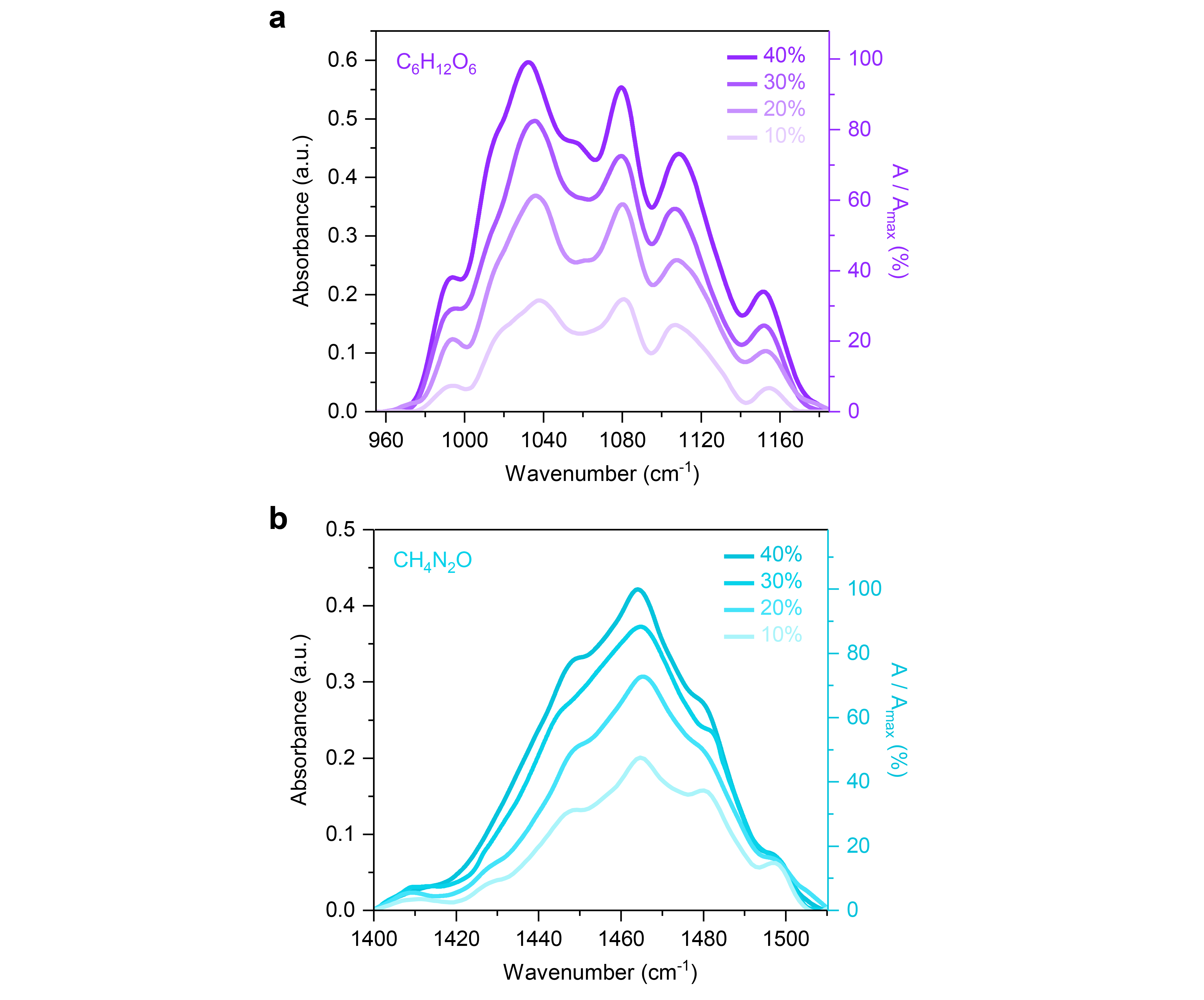


**Figure S7** Absorption spectra of **a** C_6_H_12_O_6_ and **b** CH_4_N_2_O with different concentration were detected by AST fiber with *l*_w_ = 20 mm, *d*_w_ = 50 μm. It can be observed that as the concentration of C_6_H_12_O_6_ and CH_4_N_2_O increases, the absorbance intensities of their respective characteristic peaks significantly enhance.


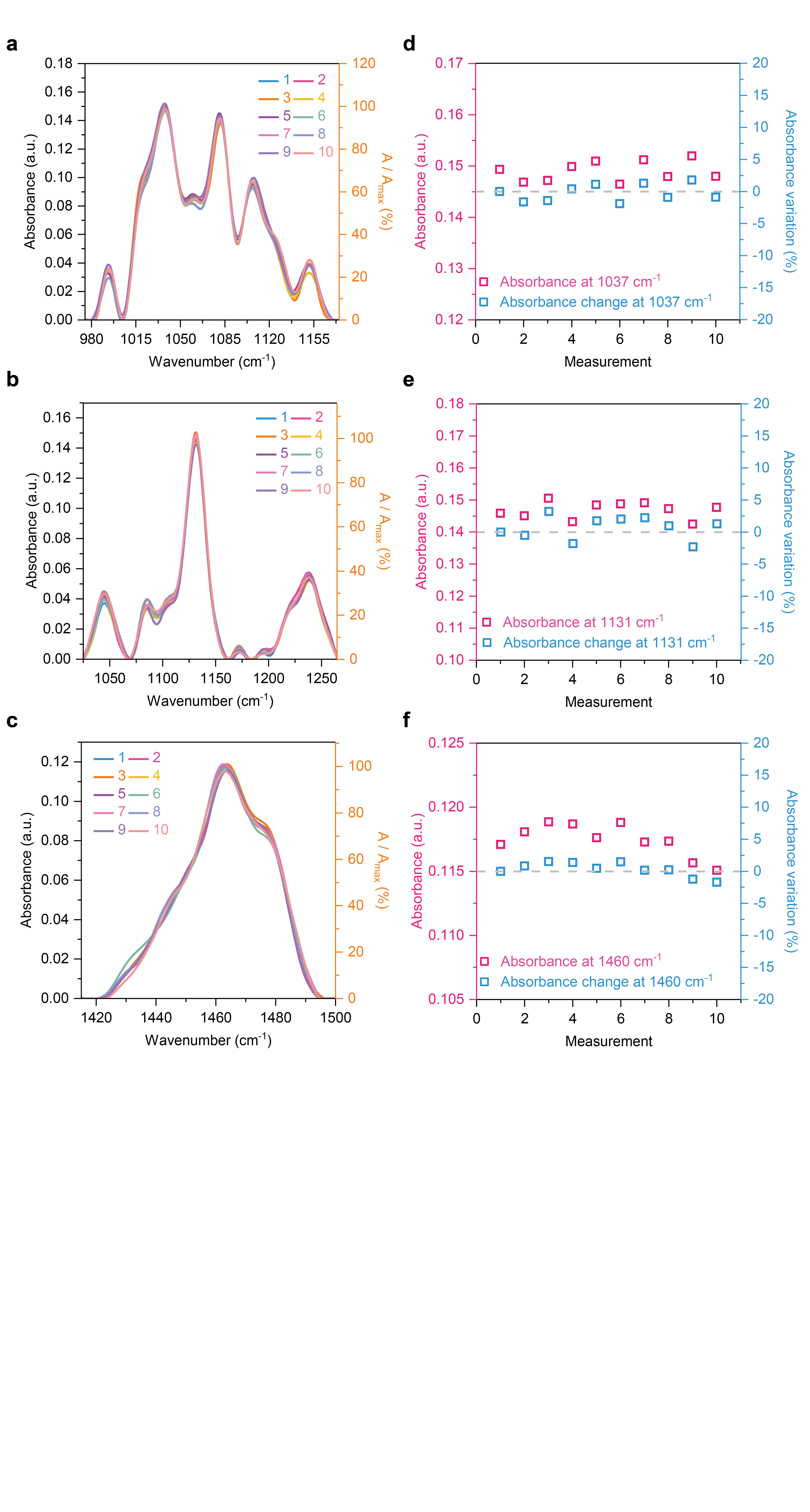


**Figure S8** Sensor accuracy. **a** Infrared spectra of 5% C_6_H_12_O_6_, **b** 5% C_3_H_6_O_3_ and **c** 5% CH_4_N_2_O were measured for 10 times. **d-f** The intensity and the variations of characteristic peaks (1037, 1131 and 1460 cm^-1^) in 10 times. As can be seen, the variations in the intensities of the characteristic peaks for C_6_H_12_O_6_, C_3_H_6_O_3_, and CH_4_N_2_O are generally within ±2%.


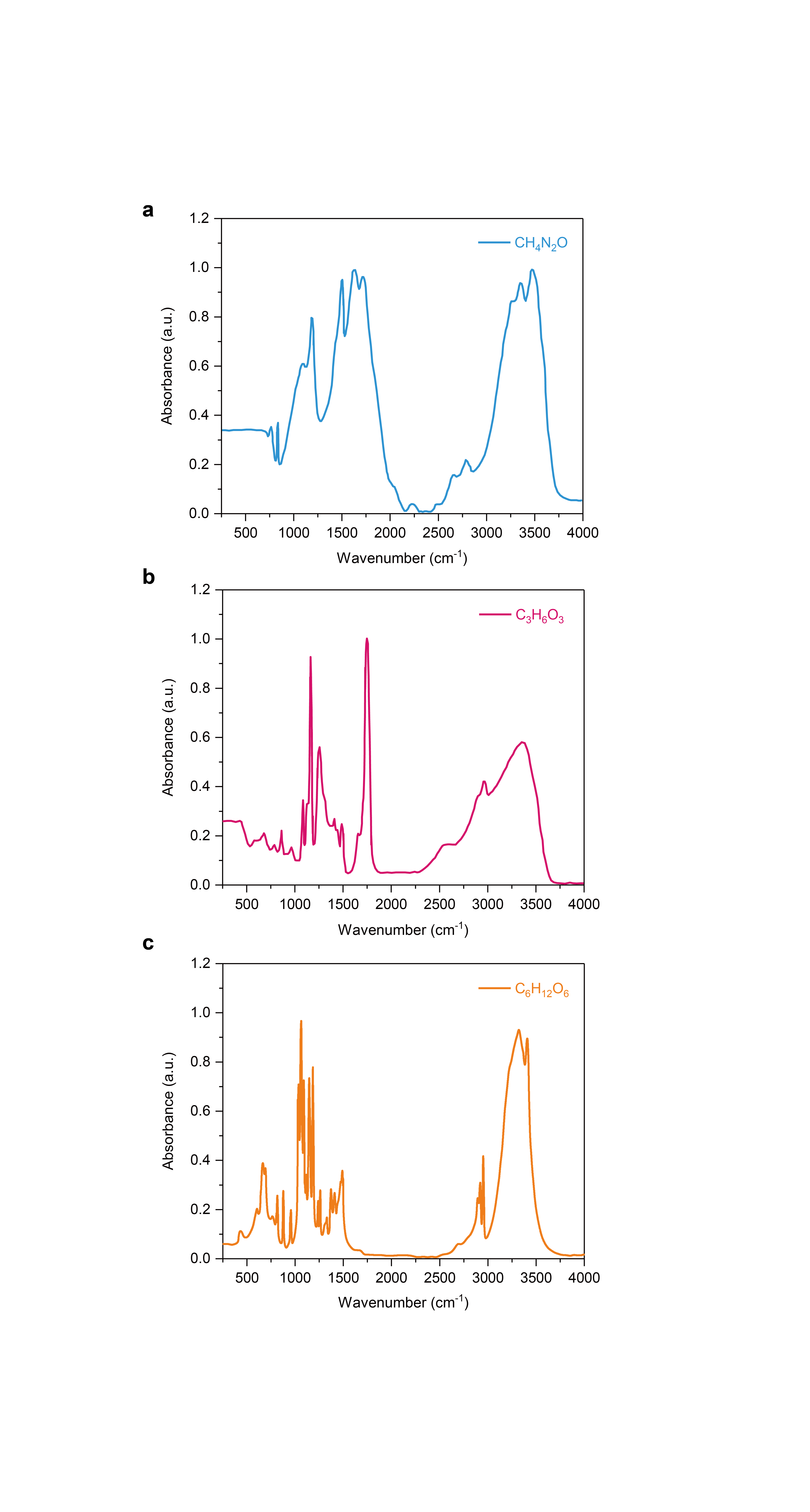


**Figure S9** The characteristic peaks of **a** CH_4_N_2_O, **b** C_3_H_6_O_3_ and **c** C_6_H_12_O_6_.


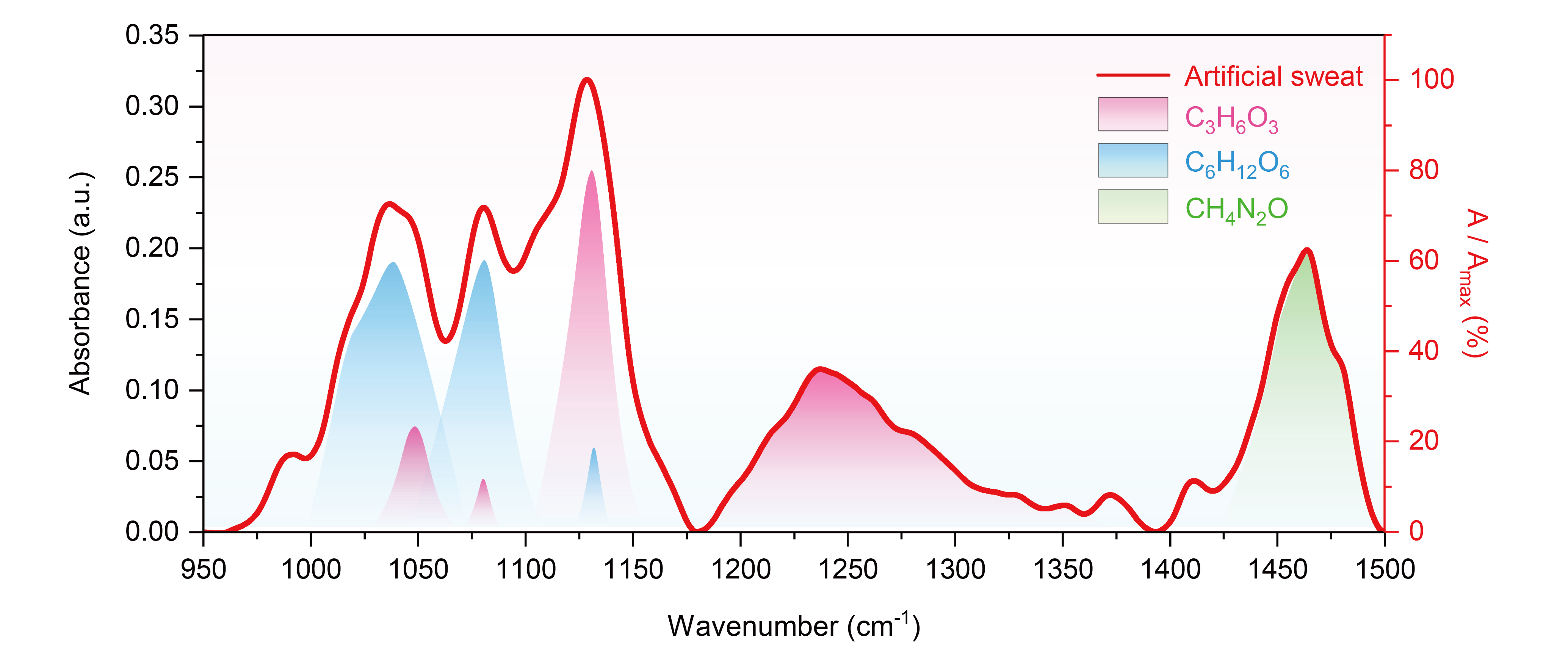


**Figure S10** MIR-FEWS absorbance spectra of artificial sweat with 10% CH_4_N_2_O, 10% C_6_H_12_O_6_ and 10% C_3_H_6_O_3_. It is evident that the peaks at 1464 cm^-1^ and 1236 cm^-1^ correspond to CH_4_N_2_O and C_3_H_6_O_3_. Therefore, the peak intensity of C_3_H_6_O_3_ at 1045, 1082m and 1133 cm^-1^ can be calculated, and obtain the characteristic peak intensity of C_6_H_12_O_6_ at 1039, 1080 and 1133 cm^-1^.


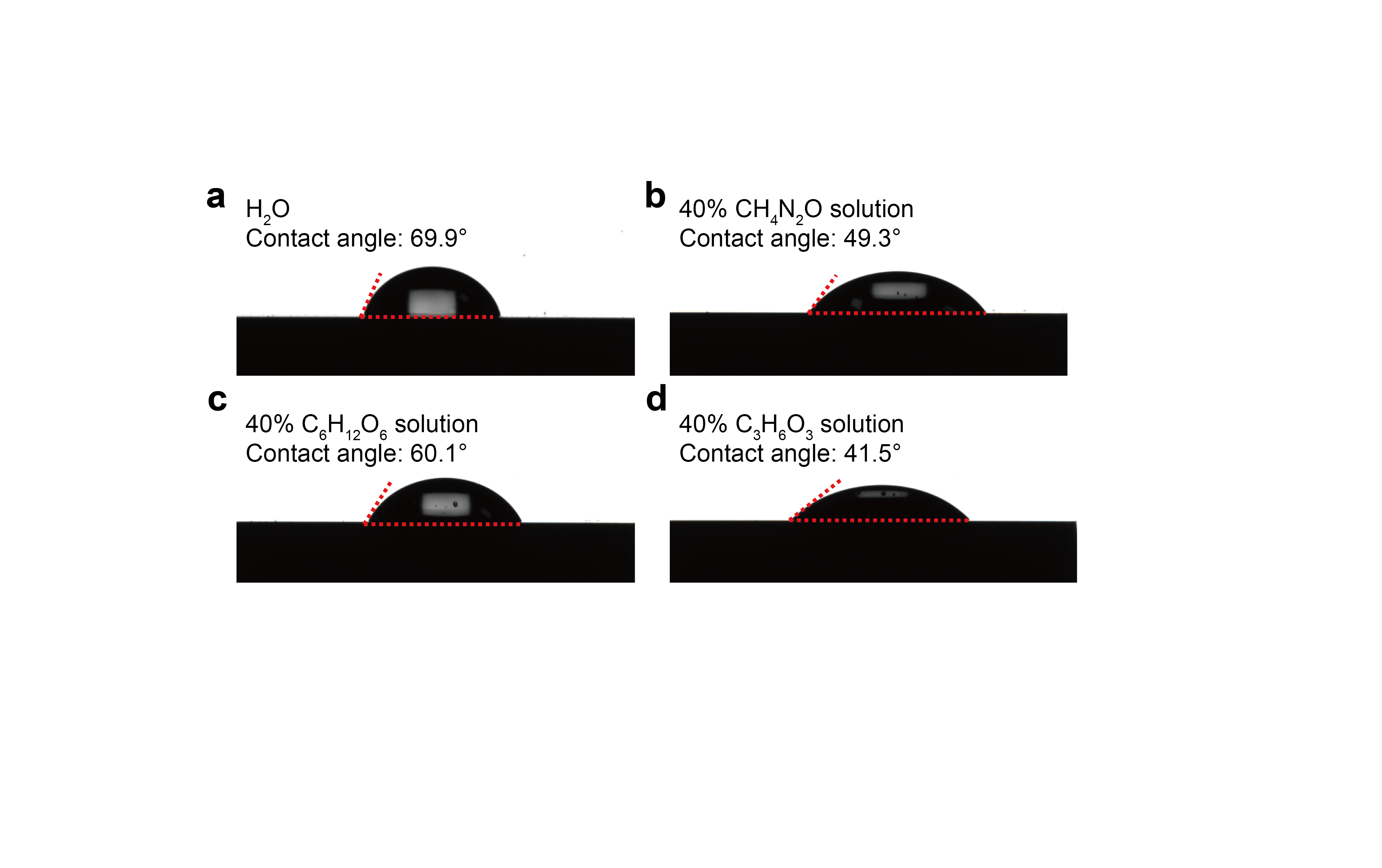


**Figure S11** Contact angle of AST glass with **a** H_2_O, **b** 40% CH_4_N_2_O, **c** 40% C_6_H_12_O_6_, and **d** 40% C_3_H_6_O_3_, respectively. The volume of each liquid is 5 μL, and the contact angles of H_2_O, CH_4_N_2_O, C_6_H_12_O_6_, and C_3_H_6_O_3_ between AST glass are 69.9°, 49.3°, 60.1°, and 41.5°, respectively.


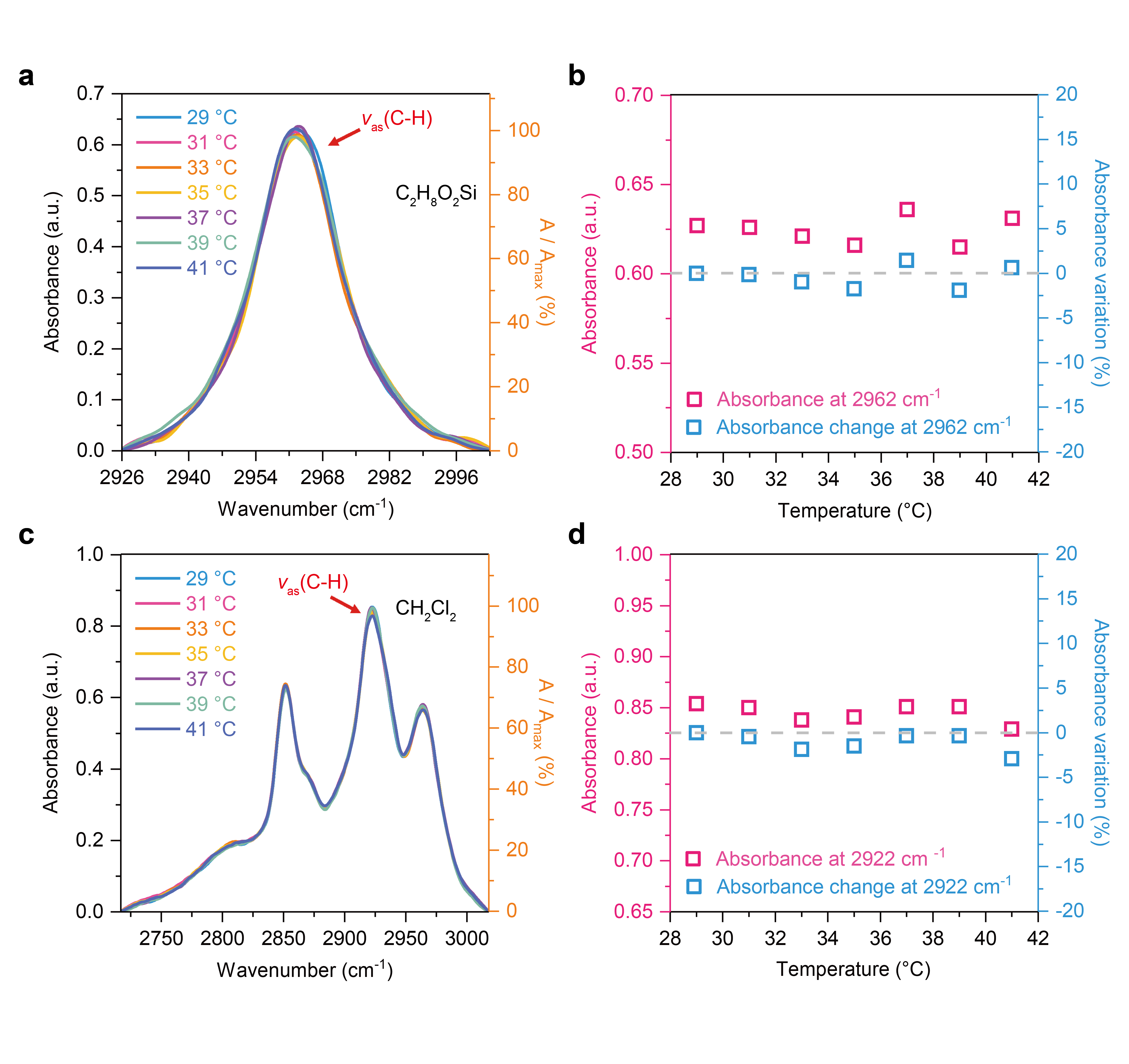


**Figure S12** Temperature effect on C_2_H_8_O_2_Si and CH_2_Cl_2_ MIR-FEWS. **a** MIR-FEWS absorbance spectra of the *v*_as_(C-H) band of C_2_H_8_O_2_Si at different temperatures. **b** *v*_as_(C-H) band absorbance variation of C_2_H_8_O_2_Si at different temperature. **c** MIR-FEWS absorbance spectra of the *v*_as_(C-H) band of CH_2_Cl_2_ at different temperatures. **d** *v*_as_(C-H) band absorbance variation of CH_2_Cl_2_ at different temperature. The result shows that with a temperature change of 12 °C, the measured change in signal absorbance is ±2%, which was within the testing error range as show in Figure S8. Thus, we can sagely neglect any temperature effect in exploiting future MIR-FEWS data.


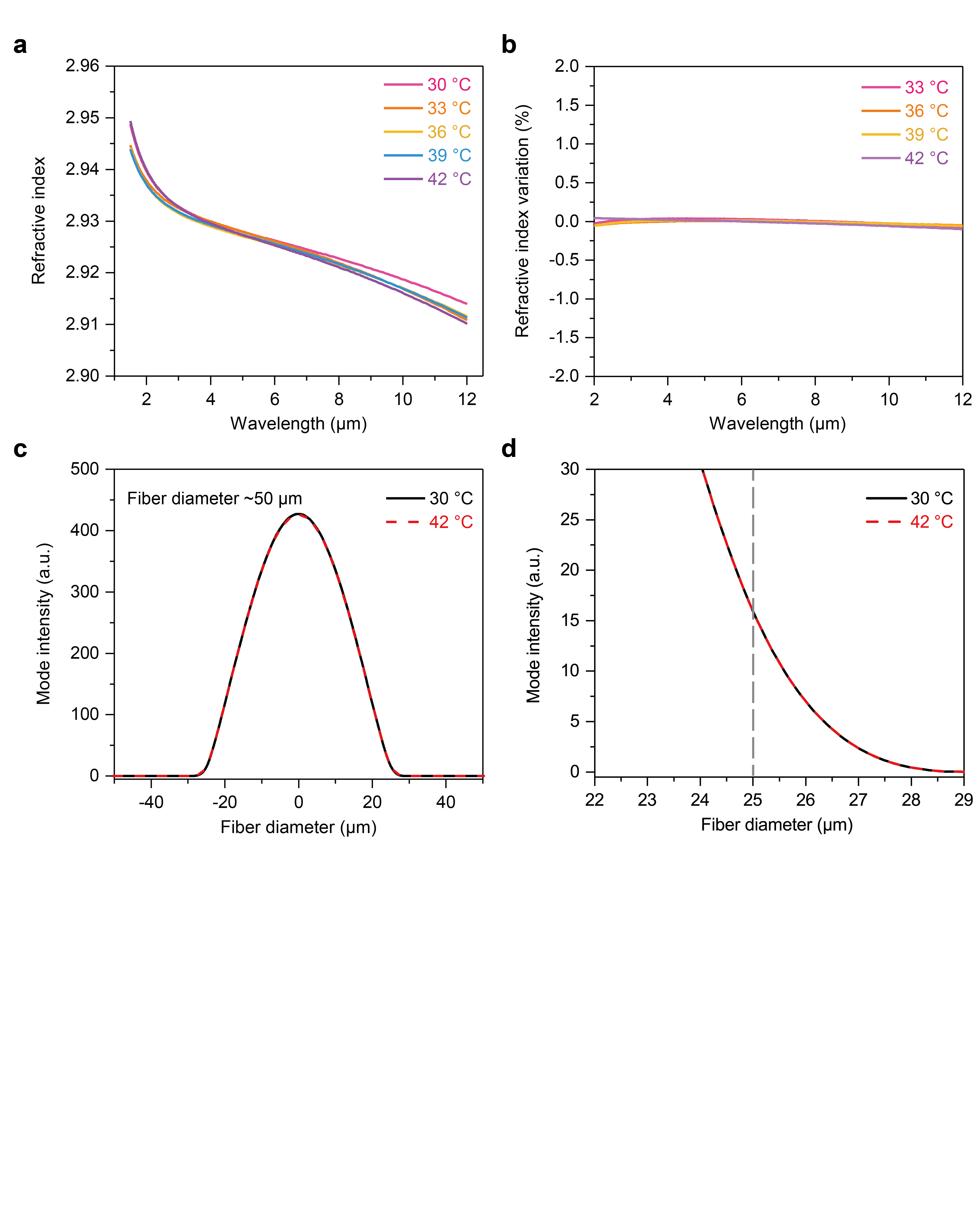


**Figure S13 a** The linear refractive index of AST glass at different temperature as a function of wavelength. **b** The refractive index variation of AST glass at different temperature as a function of wavelength. **c** Field intensity plot and **d** boundary details of AST fiber with diameters of 50 μm at 30 °C and 42 °C. The observed refractive index changes about ±0.05 at 8.85 μm. The simulation results show that under such a small refractive index variation, there is almost no change in the intensity of the fiber mode field, which allows us to neglect the temperature effect in exploiting future MIR-FEWS data.


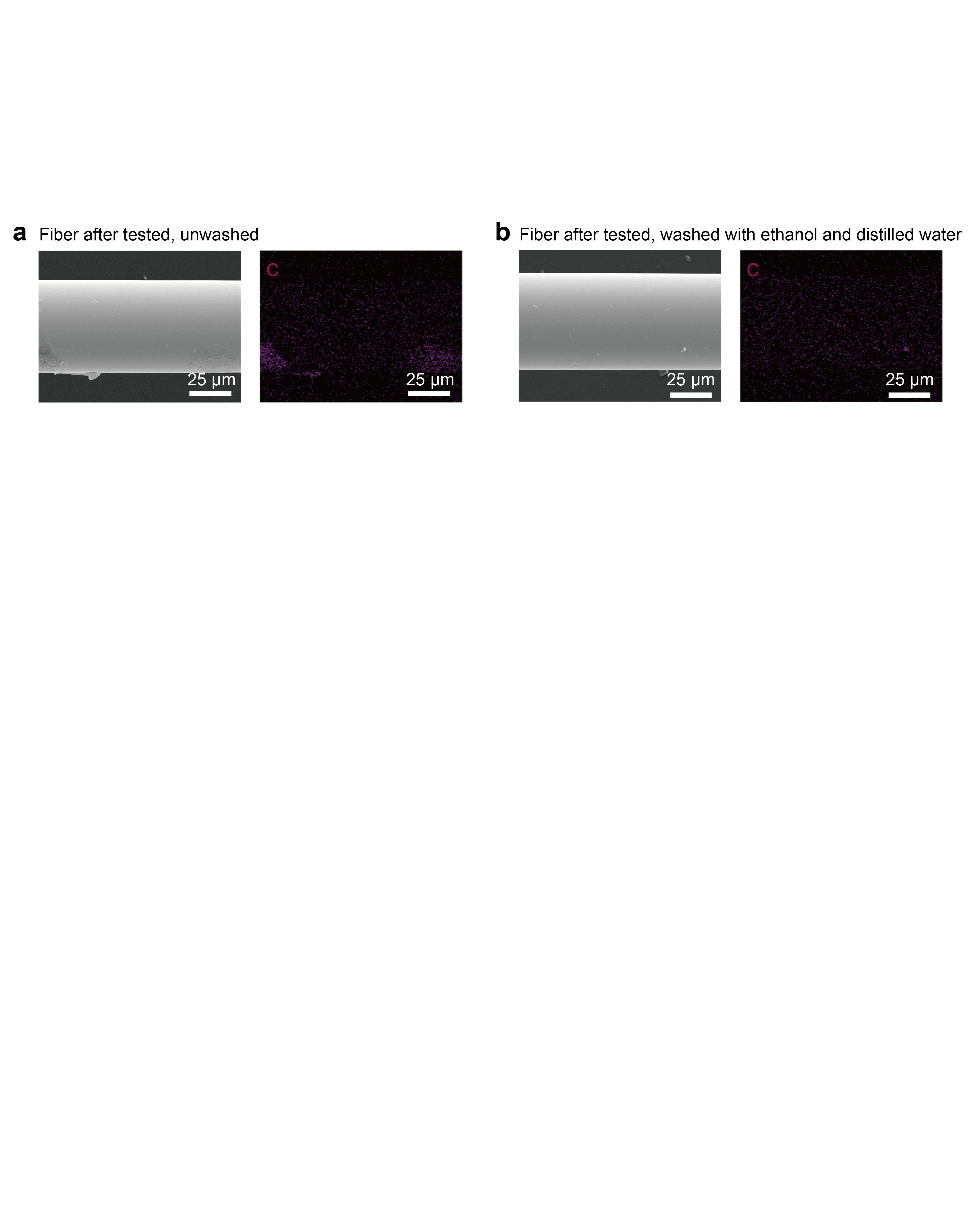


**Figure S14 a** SEM and EDS pictures of the AST fiber after tested and unwashed. **b** SEM and EDS pictures of the AST fiber after tested and washed with ethanol and distilled water. Before washing, some organic substrate aggregates are observed on the surface of the fiber. After washing, the surface of the fiber is clean without showing aggregates.


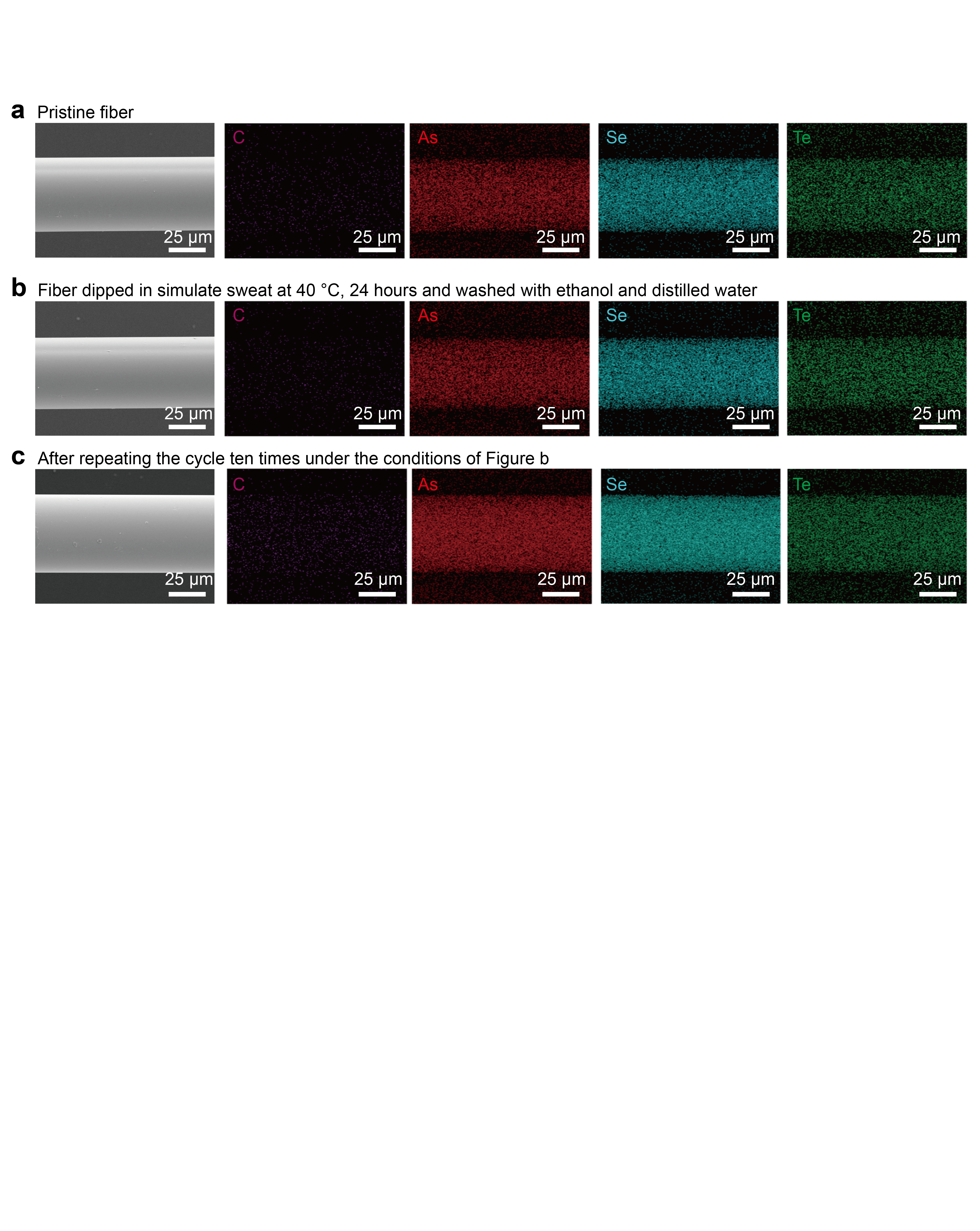


**Figure S15** SEM and EDS pictures of the AST fiber before and after test. **a** SEM and EDS images of the pristine AST fiber. **b** SEM and EDS images of the fiber dipped in simulate sweat (contains 0.5 % C_3_H_6_O_3_, 0.5% CH_4_N_2_O and 0.5% C_6_H_12_O_6_) at 40 °C. After 24 hours, the fiber is washed with ethanol and distilled water. **c** SEM and EDS images of the fiber after repeating the cycle ten times under the conditions of Figure S14b. It can be seen that no degradation is observed on the fiber surface. Furthermore, it is observed that there is no enrichment of organic substance on the fiber surface, thus confirming the stability and repeatability of the fiber in the sweat and temperature monitoring.


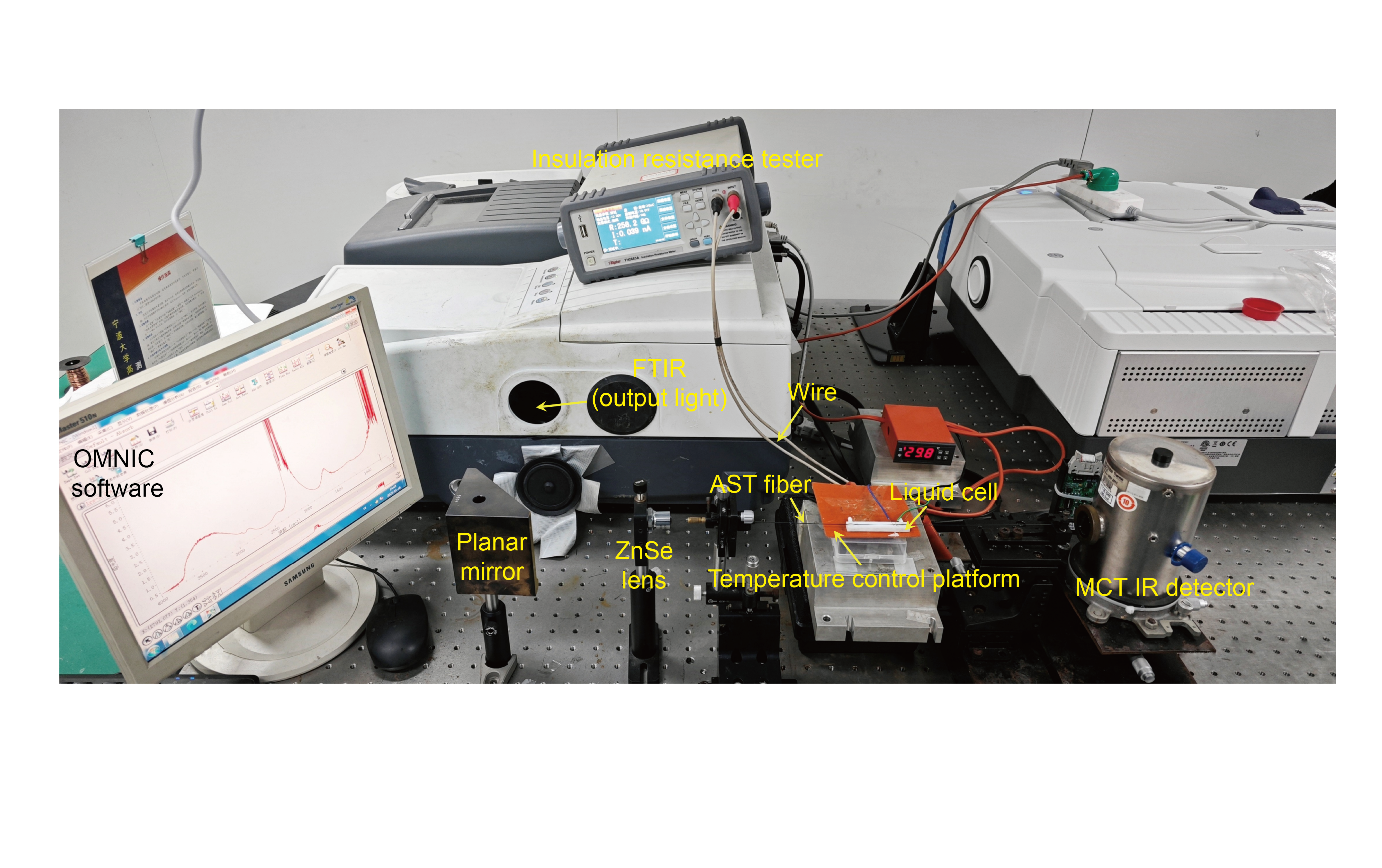


**Figure S16** Photograph of the dual-sensing platform for sweat biomarker and temperature measurements using an AST fiber.


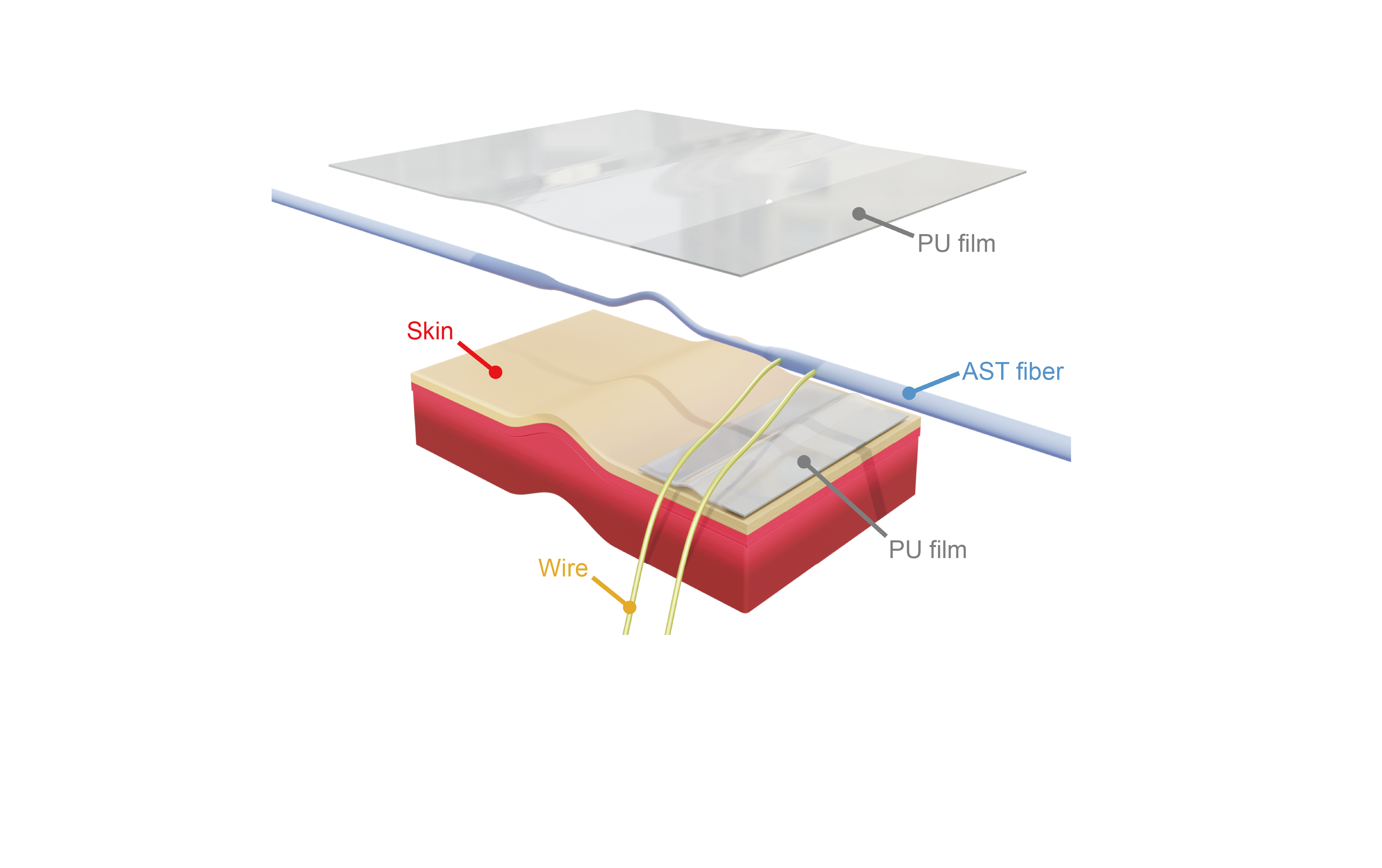


**Figure S17** Schematic diagram of AST fiber for human sweat and temperature sensing.


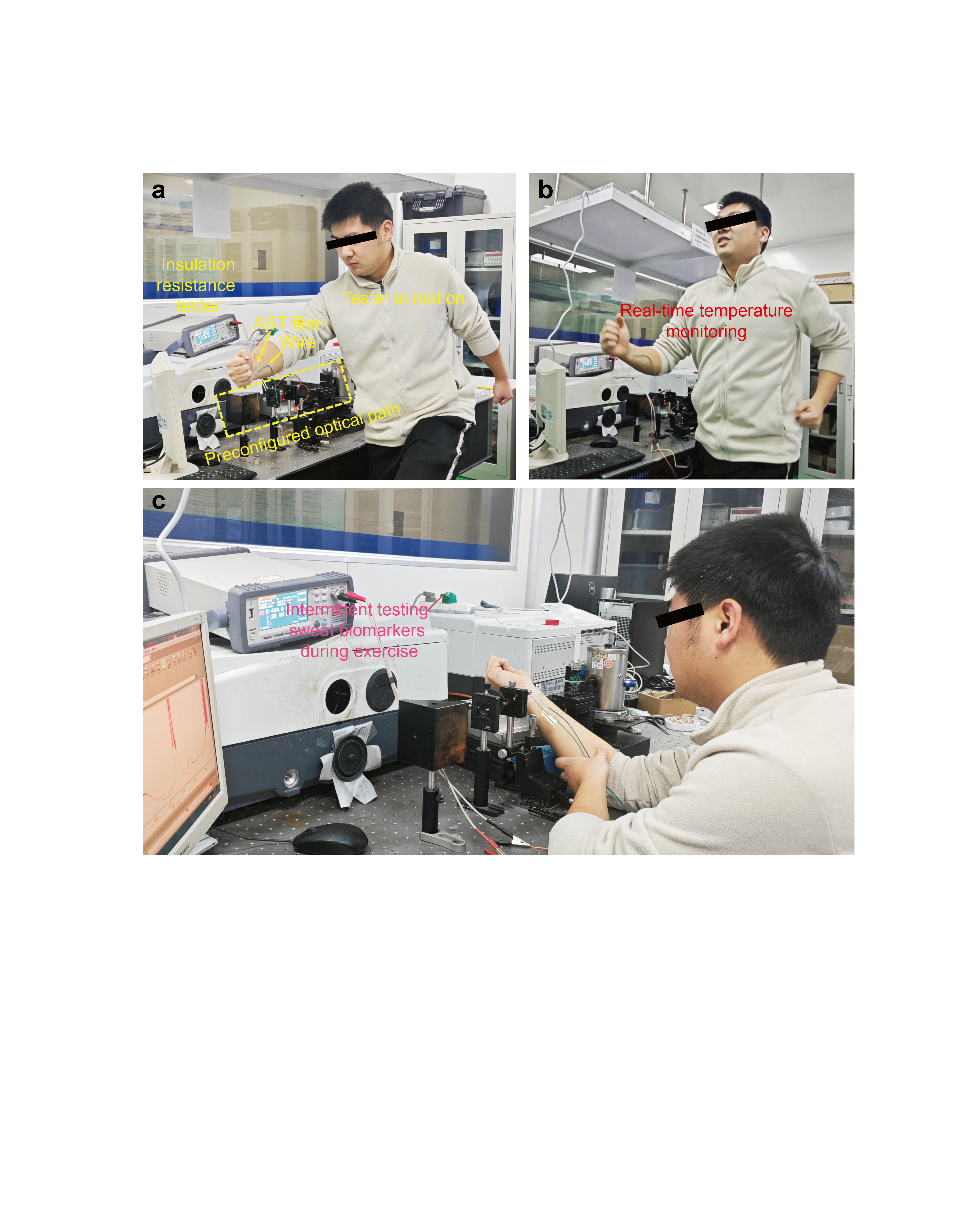


**Figure S18 a** Device connection method during exercise. **b** Real-time resistance measurement of the AST fiber during exercise. **c** Sweat biomarker information collected every 300 s.

**Table S1.** Comparison of different resistance temperature sensing materials

| Materials | Temperature range  (K) | Average \|TCR\|  (% K^-1^) | References |
| --- | --- | --- | --- |
| Ag_2_S | 303-311 | 3.63 | 1 |
| Ni | 303-313 | 0.60 | 2 |
| CNT/PEDOT: PSS | 303-313 | 0.25 | 3 |
| CNT | 307-310 | 1.35 | 4 |
| MoS_2_ | 303-313 | 2.00 | 5 |
| Pt | 304-308 | 0.16 | 6 |
| Rubber/CNT | 308-313 | 0.70 | 7 |
| CNT | 303-313 | 1.23 | 8 |
| PEDOT:PSS/MWNT | 303-313 | 0.76 | 9 |
| PEDOT: PSS/CuPc | 303-313 | 0.19 | 10 |
| Pt/constantan alloy | 303-313 | 0.02 | 11 |
| PVA/glycerol | 303-313 | 0.96 | 12 |
| Ni-Cr | 303-313 | 0.04 | 13 |
| ACNF | 303-313 | 1.52 | 14 |
| CNT | 305-317 | 0.08 | 15 |
| As_3_Se_5_Te_2_ | 303-313 | 5.84 | This work |

**Table S2.** Origin of the infrared absorption peaks of CH_4_N_2_O, C_6_H_12_O_6_ and C_3_H_6_O_3_

| **CH_4_N_2_O** | | **C_6_H_12_O_6_** | | **C_3_H_6_O_3_** | |
| --- | --- | --- | --- | --- | --- |
| Wavenumber  (cm^-1^) | Origin | Wavenumber  (cm^-1^) | Origin | Wavenumber  (cm^-1^) | Origin |
| 3483 | *v*_as_(NH) | 3402 | *v*(OH) | 2947 | *v*_s_(CH_3_) |
| 3359 | *v*_s_(NH) | 2944 | *v*_s_(CH_3_) | 2897 | *v*(CH) |
| 1715 | *v*(C=O) | 1367 | $\delta$(H-CO) | 1743 | *v*(C=O) |
| 1624 | $\delta$_s_(CN_2_) | 1319 | $\delta$(H-CO) | 1486 | $\delta$_as_(CH_3_) |
| 1496 | *v*_s_(CN) | 1260 | $\delta$(CH) | 1410 | *v*_s_(CO_2_^-^) |
| 1186 | $\rho$(NH_2_) | 1239 | $\delta$(OH) | 1253 | $v$_as_(CCO-O) |
|  |  | 1184 | $\delta$(CH) | 1155 | *v*_s_(C-O-C) |
|  |  | 1148 | *v*_s_(C-O-C) | 1086 | $\rho$(CH_3_) |
|  |  | 1116 | *v*(C-O) | 960 | *r*(CH_3_) |
|  |  | 1064 | *v*(C-O) | 861 | *v*(C-COO) |
|  |  | 1037 | *v*(C-O) |  |  |
|  |  | 952 | *v*(C-O) |  |  |

**Table S3.** C_6_H_12_O_6_, C_3_H_6_O_3_, and CH_4_N_2_O content and temperature of artificial sweat at different stages in Figure 4c

| Number | C_6_H_12_O_6_ (%) | C_3_H_6_O_3_ (%) | CH_4_N_2_O (%) | Temperature (°C) |
| --- | --- | --- | --- | --- |
| 1 | 0 | 0 | 0 | 36.5 |
| 2 | 0 | 0.3 | 0 | 37.0 |
| 3 | 0.1 | 0.3 | 0.1 | 37.5 |
| 4 | 0.3 | 0.5 | 0.3 | 38.0 |
| 5 | 0.5 | 0.8 | 0.3 | 38.5 |
| 6 | 1 | 1 | 0.5 | 39.0 |
| 7 | 1.5 | 1.5 | 1 | 39.5 |
| 8 | 2 | 2 | 1.5 | 40.0 |

**Supplemental Movie Legends**

**Movie S1.**

Simultaneous monitoring of biomarkers (water and ethanol) and temperature using AST fiber

**Movie S2.**

Simultaneous monitoring of biomarker (lactic acid) and temperature using AST fiber

**References**

1. Zhao, X. F. et al*.* A Fully Flexible Intelligent Thermal Touch Panel Based on Intrinsically Plastic Ag_2_S Semiconductor. *Advanced Materials* **34**, 2107479 (2022).

2. Shin, J. et al*.* Sensitive wearable temperature sensor with seamless monolithic integration. *Advanced Materials* **32**, 1905527 (2020).

3. Harada, S. et al. Fully printed flexible fingerprint-like three-axis tactile and slip force and temperature sensors for artificial skin. *ACS Nano* **8**, 12851-12857 (2014).

4. Kuzubasoglu, B. A. et al. Wearable temperature sensor for human body temperature detection. *Journal of Materials Science: Materials in Electronics* **32**, 4784-4797 (2021).

5. Daus, A. et al*.* Fast-response flexible temperature sensors with atomically thin molybdenum disulfide. *Nano Letters* **22**, 6135-6140 (2022).

6. Yoo, T. et al*.* The real-time monitoring of drug reaction in HeLa cancer cell using temperature/impedance integrated biosensors. *Sensors and Actuators B: Chemical* **291**, 17-24 (2019).

7. Lin, M. Z. et al*.* A high‐performance, sensitive, wearable multifunctional sensor based on rubber/CNT for human motion and skin temperature detection. *Advanced Materials* **34**, 2107309 (2022).

8. Wu, R. H. et al*.* Silk composite electronic textile sensor for high space precision 2D combo temperature-pressure sensing. *Small* **15**, 1901558 (2019).

9. Oh, J. H. et al*.* Fabrication of high-sensitivity skin-attachable temperature sensors with bioinspired microstructured adhesive. *ACS Applied Materials & Interfaces* **10**, 7263-7270 (2018).

10. Li, J. et al*.* High-resolution temperature sensor fabricated with composed PEDOT: PSS/CuPc for electronic skin. *Sensors and Actuators A: Physical* **363**, 114706 (2023).

11. Hua, Q. L. et al*.* Skin-inspired highly stretchable and conformable matrix networks for multifunctional sensing. *Nature Communications* **9**, 244 (2018).

12. Gu, J. F. et al*.* Multifunctional poly (vinyl alcohol) nanocomposite organohydrogel for flexible strain and temperature sensor. *ACS Applied Materials & Interfaces* **12**, 40815-40827 (2020).

13. Eom, T. H. & Han, J. I. The effect of the nickel and chromium concentration ratio on the temperature coefficient of the resistance of a Ni-Cr thin film-based temperature sensor. *Sensors and Actuators A: Physical* **260**, 198-205 (2017).

14. Lee, J. H. et al*.* Flexible temperature sensors made of aligned electrospun carbon nanofiber films with outstanding sensitivity and selectivity towards temperature. *Materials Horizons* **8**, 1488-1498 (2021).

15. Chhetry, A. et al*.* Black phosphorus@ laser‐engraved graphene heterostructure‐based temperature-strain hybridized sensor for electronic‐skin applications. *Advanced Functional Materials* **31**, 2007661 (2021).
